# A Theory-Based Longitudinal Investigation Examining Predictors of Self-Harm in Adolescents With and Without Bereavement Experiences

**DOI:** 10.3389/fpsyg.2020.01153

**Published:** 2020-06-03

**Authors:** Laura del Carpio, Susan Rasmussen, Sally Paul

**Affiliations:** ^1^School of Psychological Sciences & Health, University of Strathclyde, Glasgow, United Kingdom; ^2^School of Social Work & Social Policy, University of Strathclyde, Glasgow, United Kingdom

**Keywords:** adolescence, self-harm, suicide, bereavement, theory, IMV model

## Abstract

**Background:**

Research has demonstrated that exposure to suicide can lead to increased vulnerability for self-harm or suicide. As a result, ideation-to-action models of suicide (e.g., the Integrated Motivational-Volitional Model of Suicide; IMV) recognise exposure as a significant risk factor which may be implicated in the translation of thoughts into actions. However, few studies have tested this theoretical link explicitly within an adolescent population, and examined how it compares to other types of bereavements.

**Methods:**

A 6-month prospective questionnaire study was conducted with 185 Scottish adolescents aged 11–17 (115 adolescents also completed the questionnaire at follow-up). The questionnaire included measures on experiences with bereavement and lifetime engagement in self-harm, as well as measures of defeat, entrapment, social support, coping, and other psychological variables.

**Results:**

At baseline, 11% of young people reported exposure to a suicide death, and 62% to a non-suicide death. In addition, 21% of pupils reported ever engaging in self-harm, while 24% had experienced self-harm ideation without engaging in it. Cross-sectional multivariate logistic regressions showed that maladaptive coping, family social support, glorifying/normalising beliefs about suicide, and family self-harm were significantly associated with self-harm group membership (control, ideation, or enactment groups). At follow-up, 9% of pupils reported exposure to a suicide death and 11% to a non-suicide death for the first time. A total of 29% of the sample reported self-harm at T2 (8% of participants for the first time), and 23% reported self-harm ideation without engaging in it. Multivariate analyses found that stigmatising beliefs about suicide, glorifying/normalising beliefs about suicide, and self-harm ideation at baseline were the only variables to predict self-harm group membership prospectively. Bereavement experiences, whether by suicide or non-suicide, did not predict self-harm group status at baseline nor at follow-up.

**Conclusions:**

This study provides support for the validity of a theoretical model of suicide, even though predictive ability over the 6-months period was limited. Although difficulties with recruitment may have limited the statistical power, this study provides insight into the prevalence and experiences of suicide bereavement among adolescents and the factors related to the onset and maintenance of self-harm.

## Introduction

Suicide is a major public health problem across the globe, representing the second leading cause of death among young people from 15 to 29 years old worldwide (World Health Organization [WHO], 2014). The most recent data from Scotland reveals that 784 people took their own lives in 2018 (Scottish Public Health Observatory, 2019). The rate of young people aged 15–24 dying by suicide in 2018 was at its highest since 2007, at a rate of 15.1 per 100,000 population, an increase of over 50% since the previous year.

Research has found that over half of young people under 20 years old who died by suicide had a history of self-harm (University of Manchester, 2017), and evidence consistently shows that one of the strongest predictors of suicide is self-harm, irrespective of the intention (Hawton et al., 2012). Research has also frequently demonstrated that the vast majority of young people who self-harm do not present to hospital, and consequently it is important to go beyond clinical studies to examine the prevalence and causation of adolescent self-harm (Geulayov et al., 2018).

In Scotland, research examining self-harm within community samples suggests that up to 14% of young people have engaged in (enacted) self-harm (O’Connor et al., 2009b, 2012), and a further 22.8% have thought (ideated) about self-harm (Russell et al., 2018). These findings are comparable to other studies in the United Kingdom (Hawton et al., 2002; McMahon et al., 2010). This research highlights that self-harm is a significant issue affecting young people, and understanding the risk and protective factors for self-harm is therefore imperative to reduce the potential negative outcomes associated with this behaviour. National suicide prevention strategies also recognise self-harm as a crucial component of suicide prevention (Scottish Government, 2018b; HM Government, 2019), emphasising the need for evidence-based interventions to reduce self-harm at the community level.

### Suicide Bereavement: Risk Factor for Self-Harm and Suicide

Exposure to the fatal and non-fatal self-harm of others has been cited as a predictor of future suicidal or self-harming thoughts and behaviours (SSHTBs; O’Connor et al., 2009a; Hawton et al., 2012; O’Connor and Nock, 2014; Mars et al., 2019a). Regarding non-fatal self-harm, De Leo and Heller (2004) analysed responses from 3,757 secondary school pupils as part of the Child and Adolescent Self-Harm in Europe (CASE) study. They found that exposure to the self-harm of others significantly predicted one’s own self-harm; self-harm among friends was associated with a higher odds of self-harm than self-harm among family members (*OR* = 4.07 vs. 3.22). Similarly, McMahon et al. found that approximately one third of adolescents in Ireland had been exposed to a friend or family member’s self-harm or suicide, and these individuals were almost eight times more likely to report self-harm compared to those without such exposure (McMahon et al., 2013). Evidence of the association between fatal self-harm (i.e., suicide) and adverse outcomes is less clear. This is partly due to the uncertainty about how many adolescents are dealing with a suicide bereavement. It is known that bereavement is a common experience among young people; Harrison and Harrington (2001) found that up to 77.6% of adolescents from 11 to 16 years old in England had experienced the death of a first or second-degree relative or close friend. In Great Britain, one study found that 3.5% of children and adolescents (5–16 years old) reported being bereaved by a parent or sibling, 6.3% being bereaved of a friend, and 0.3% of both (Fauth et al., 2009). However, no known studies have determined the proportion of young people in Scotland who are bereaved by suicide. This is significant given that many people are known to be affected by each suicide, with estimates ranging from 6 people (Shneidman, 1972), to 10 (Andriessen and Krysinska, 2012), 80 (Berman, 2011) and up to 135 (Cerel et al., 2018) individuals affected by every suicide. A recent meta-analysis predicted that 21.83% of individuals are exposed to suicide at some point in their lives, with 4.31% in the past year (Andriessen et al., 2017). Identifying how many adolescents in Scotland are bereaved by suicide will therefore aid in understanding its impact.

The sequelae of bereavement or exposure to a suicide (i.e., experiencing the suicide death of someone important to oneself, such as a family member or friend) may extend beyond that of other types of deaths. Those bereaved by suicide may face a number of additional challenges as a result of the manner of death, including increased perceptions of rejection, shame, stigma, blame, and a need to conceal the cause of death (see Sveen and Walby, 2008 for a review). An elevated risk of mental and physical health problems, particularly depression, anxiety, and posttraumatic stress disorder (Pitman et al., 2014; Erlangsen and Pitman, 2017), as well as prolonged grief reactions (Young et al., 2012), have also been indicated. Empirical evidence of increased risk of SSHTBs is less consistent, and may be the result of varying methodological approaches, the quality or type of relationships being investigated, or age groups in question.

Within the adolescent literature, family suicide is a strong risk factor for subsequent SSHTBs. Guldin et al. (2015) showed that suicide bereaved children had a greater incidence of suicide than children bereaved by accidental or other deaths, and the risk persisted for decades. Similar patterns were reported by Wilcox et al. (2010) and Kuramoto et al. (2013), who highlight childhood and adolescence as a vulnerable time. It is also suggested that parental sex may play an important role, with adolescents more likely to die by suicide if their same-sex parent died by suicide (Cheng et al., 2014), and the risk is independent of family history of psychiatric illness (Qin et al., 2002). Despite evidence to suggest that family suicide is a risk factor for adolescent SSHTBs, less is known about the influence of other non-familial suicide deaths. Cross-sectional research (e.g., Pirelli and Jeglic, 2009) has been more likely to find an association between peer exposure and suicidality, compared to longitudinal research (Andriessen et al., 2016). However, there is a dearth of control-group studies looking at adolescents’ experiences with bereavements outside of the immediate family, and prospective research in this area is particularly scarce. Longitudinal work is valuable in that it allows for the prediction of future behaviours, and thus provides particularly strong evidence for identifying risk factors for self-harm (Ribeiro et al., 2016).

### Suicide Theory: The IMV Model of Suicidal Behaviour

There has been a call for more theoretically driven work in order to develop a cumulative evidence base which can inform suicide intervention development going forward; however, at the moment there is a paucity of theoretically informed work in this field. Theoretical conceptualisations recognise exposure to suicide as a risk factor for SSHTBs. The Integrated Motivational-Volitional (IMV) Model of Suicide (O’Connor, 2011; O’Connor and Kirtley, 2018) offers a framework to understand the development of thoughts and their translation into behaviours, which are seen as distinct processes within an ideation-to-action framework (Klonsky and May, 2015). Applicable to self-harm as well as suicide, it proposes that a convergence of biological, psychological, and environmental risk and protective factors contribute to suicide. Importantly, factors, which predict the formation of suicidal ideation, are thought to be distinct from those which predict behavioural enaction. Background vulnerability factors and triggering events are said to predispose a person to feel defeated and humiliated. This can result in perceptions of entrapment, which may result in suicidal thoughts. These transitions are facilitated by Threat to Self (TSM) and motivational moderators (MM), which increase/decrease the likelihood of a person moving between each of these key stages. A number of factors, or volitional moderators (VMs), subsequently increase or decrease the likelihood that a person will go on to engage in suicidal behaviours after experiencing thoughts.

Within the IMV model, exposure to the SSHTBs of others is recognised as a key volitional factor in the transition from ideation to attempts. The model proposes that people who engage in self-harm behaviours are more likely to have been exposed to self-harm or suicide than those who only experience thoughts of self-harm or those with no history of self-harm. Research specifically testing the IMV model finds support for this notion (Dhingra et al., 2015; Mars et al., 2019b), although the evidence base for adolescent experiences of losing someone to suicide is limited. Studies investigating this relationship would also benefit from taking into account other factors which impact adjustment after a loss.

One factor purportedly relevant to the development of SSHTBs within the TSMs is one’s coping style. Research has shown that coping ability is related to levels of suicidality (after controlling for depression), and may interact with levels of defeat and entrapment to elevate or reduce risk of suicide (Gooding et al., 2015). Studies also demonstrate that suicide bereaved youth engage in increased risk taking behaviours to cope with a loss, such as alcohol and drug misuse and risky sexual behaviours (Bartik et al., 2013a), and may utilise more avoidant coping strategies, such as distraction and social diversion (Bartik et al., 2013b). Relatedly, self-esteem has been associated with vulnerability to suicide, where low levels of self-esteem were associated with high suicide probability (measured through the constructs of hopelessness, suicidal ideation, hostility, and negative self-evaluations) even after controlling for depression (Gooding et al., 2015). Among adolescents, Seguin et al. (2004) showed that individuals who attempted suicide and experienced suicidal ideation differed from controls (with no history of self-harm), but not each other, on levels of self-esteem. Self-esteem was also predictive of subsequent depression among adolescents bereaved by parental death (Brent et al., 2009), suggesting it may buffer the impact of a bereavement on young people.

The social context of an individual is recognised within the proposed MMs of the model. Studies have consistently found that social support is associated with suicide risk (Kleiman and Liu, 2013; O’Connor and Nock, 2014), as well as adjustment after a death (Andriessen et al., 2015). Perceptions of loneliness and altered social roles may explain why suicide bereavement leads to increased vulnerability for self-harm (Pitman et al., 2014). In addition, attitudes and stigma surrounding suicide (as another possible MM) have been highlighted as important in mental health and suicide outcomes. Bartik et al. (2015) found that young people who had experienced a suicide bereavement were less likely than a general population sample to view suicide as resulting from isolation and depression, and more likely to view those who die by suicide with stigma and in glorifying or normalising terms. Ultimately, endorsing stigmatising beliefs about suicide may prevent individuals from talking about it or seeking help when needed, and therefore increase their vulnerability to adverse outcomes.

To further our understanding of the link between bereavement experiences and subsequent self-harm behaviours, research is needed to understand the interrelationships between bereavement and other factors which may be relevant to self-harm. By providing testable hypotheses, theory-based investigations can ultimately guide suicide prevention efforts by providing an evidence-base for interventions that reduce known risk factors for self-harm. Effective postvention, or the support offered to people bereaved by suicide, is an area that requires further empirical research given that it is a key target for suicide prevention and policy (Scottish Government, 2018b; Andriessen et al., 2019). As an emerging theoretical model, the IMV model requires further testing, particularly with diverse populations and age groups, to better understand the mechanisms underpinning suicide. Its evaluation would be of specific value to researchers, practitioners and policymakers given its potential to inform practice.

### The Current Research Study

While evidence points to suicide bereavement being a risk factor for subsequent SSHTBs, this association requires specific testing among adolescent populations. Research needs to quantify how many young people are bereaved by suicide, and how this experience is related to other factors important in the development of SSHTBs. Discussion about why exposure to suicide may increase the likelihood of self-harm behaviours in some but not others may be usefully informed by reference to theories such as the IMV. Longitudinal research is also necessary, as much of the literature to date has been cross-sectional. Prospective work is required, which can aid in identifying future targets for community-based interventions for self-harm.

The current study aimed to explore whether exposure to suicide or other deaths is associated with self-harm behaviours cross-sectionally, and longitudinally over a 6-month follow-up, and examines whether 11 relevant IMV model variables may be important in this relationship. This was achieved by examining factors highlighted in the existing literature as being relevant to the emergence of suicidal or self-harming behaviours among adolescents, within the context of an ideation-to-action framework. Specifically, the following hypotheses rooted in the IMV model were proposed:

(a) Ideation and enactment groups will differ from controls, but not each other, on motivational phase measures (defeat, entrapment, social support, coping, self-esteem, and attitudes to suicide).

(b) Ideation and enactment groups will differ from controls, as well as each other, on volitional phase measures (exposure to suicide deaths, family self-harm, and friend self-harm). Exposure to non-suicide deaths will not differentiate groups (control, ideation, or enactment).

In order to examine cross-sectional associations as well as longitudinal predictions, analyses were conducted twice, with variables predicting self-harm outcomes at baseline (T1) as well as approximately 6-months later (T2).

## Materials and Methods

### Participants

A total of 185 pupils (aged 11–17, *M* = 13.16, *SD* = 1.49) were recruited at T1 from nine secondary schools across Scotland. This sample consisted of individuals retained after removing participants with >50% missing data (*n* = 2) or who did not provide data on any of the SSHTB outcome measures (*n* = 22), and including participants from T2 who only provided data once and not at baseline (i.e., were absent at the first time point, or baseline data was removed due to missingness but T2 questionnaire was complete; *n* = 15). Of the T1 sample, 85 stated they were male, 97 female, 2 other, and 1 did not respond. Approximately half were in Year 1 (predominantly aged 12–13) of secondary school (*n* = 91, 49.2%) and described their ethnicity as White (*n* = 164, 88.65%), consistent with the last Scottish Census (96.1%; National Records of Scotland, 2011). The percentage of pupils entitled to free school meals, as a proxy measure of Socioeconomic Status (SES), ranged from 4.74 to 20.99% between schools (*M* = 14.29, *SD* = 5.19), slightly lower than previous Scottish studies (e.g., mean of 17.8% in Russell et al., 2018), though comparable to the national average of 14.4% (Scottish Government, 2018a).

One hundred and fifteen individuals (aged 12–18, *M* = 13.65, *SD* = 1.52; 46 male, 67 female, 2 other) provided data for T2, which could be matched to corresponding baseline data. This sample was retained after removing data from respondents whose T2 participant identifier codes could not be confidently matched to their baseline data (*n* = 31), who had >50% missing data (*n* = 3), or who did not respond to any of the outcome measures (*n* = 5).

The retention rate of 62.16% is similar to other longitudinal studies using adolescent samples (Boergers and Spirinto, 2003; O’Connor et al., 2009a; Hasking et al., 2013, 2015; Rasmussen et al., 2016).

### Measures

An anonymous self-report questionnaire was created using measures selected on the basis of previous literature, which have been used, or deemed appropriate for use, with adolescents. Only those which are pertinent to the current study are reported here; a full list of measures used can be obtained from the authors.

#### Demographic and Control Variables

##### Demographic characteristics

Demographic characteristics included age, gender (male/female/other), and ethnicity. The SES of participants was determined by the percentage of pupils in their school entitled to free school meals, as reported by official Scottish Government (2018a), which has been used as a measurement of SES in previous schools-based research (O’Connor et al., 2009b; Russell et al., 2017).

##### Depression

Depressive symptoms were assessed using the Short Mood and Feelings Questionnaire (SMFQ; Angold et al., 1995), which consists of 13 items describing how a person may have felt or acted in the past 2 weeks. Responses are given on a 3-point scale from 0 (not true) to 2 (true), e.g., “*I thought I could never be as good as other kids.*” Internal consistency is high in the published literature (e.g., α = 0.85; Angold et al., 1995), and was 0.93 in this study.

##### Anxiety

The Generalised Anxiety Disorder (GAD-7; Spitzer et al., 2006) scale was used to measure symptoms of anxiety, and can be used as a screening tool for generalised anxiety disorder. Seven items describe common anxiety symptoms, and participants are asked to respond whether they have felt bothered by the problems over the last 2 weeks, using a 4-point scale from 0 (not at all) to 3 (nearly every day), e.g., “*Becoming easily annoyed or irritable.*” Cronbach’s α was 0.92 in this study, comparable to previous research (e.g., α = 0.92; Spitzer et al., 2006).

#### Self-Harm and Suicidal Ideation or Behaviours

##### Self-harm

Self-harm was measured using five items from the CASE study questionnaire (Hawton et al., 2006). The original questionnaire was developed through an international collaboration of researchers and experts in self-harm and school-based research, and extensively piloted before use with adolescents across several European countries and Australia. Participants in this study were asked, “*Have you ever deliberately taken an overdose* (e.g., *of pills or other medication*) *or tried to harm yourself in some other way* (*such as cut yourself*)*?*” Those responding ‘Yes, once’ or ‘Yes, more than once’ comprised the “Enactment” group.

##### Self-harm ideation

Non-suicidal self-injury (NSSI) thoughts were assessed using an item from the Self-Injurious Thoughts and Behaviours Interview (SITBI; Nock et al., 2007), namely, “*Have you ever had thoughts of purposely hurting yourself without wanting to die?* (*for example, cutting or burning*).” Suicidal thoughts were assessed with the SITBI item, “*Have you ever had thoughts of killing yourself?*” A participant who answered ‘Yes’ to either NSSI or suicidal thoughts, and ‘No’ to the self-harm behaviours question, was considered to be in the “Ideation” group used for all further analyses. Therefore, this group consisted of all individuals with previous thoughts of self-harm or suicide, irrespective of their intent or motivation (similar to the enactment group with regards to the intent or motivation of their behaviours). Individuals responding “No” to all self-harm behaviours and NSSI or suicidal thoughts questions were considered “Controls” with no history of self-harm thoughts or acts. The SITBI has been shown to be a valid and reliable measure of a wide range of self-harm related constructs (Nock et al., 2007).

Whether a participant had engaged in first-time or repeat self-harm or self-harm ideation over the study period could be ascertained by changes in their responses to these questions across the two time-points. Although self-harm group was the outcome measure in the main analyses (T1 self-harm group for cross-sectional analyses, and T2 self-harm group for prospective analyses), self-harm ideation at baseline was also inputted as a predictor variable within the motivational phase test in the prospective analyses, given research suggesting it is a strong predictor of future self-harm (O’Connor, 2011; Ribeiro et al., 2016; O’Connor and Kirtley, 2018).

#### Motivational Phase Variables

##### Defeat

Defeat was assessed via the Defeat Scale (Gilbert and Allan, 1998), measuring perceptions of failed struggle and loss of rank or status experienced over the last week. Participants respond to 16 items using a 5-point scale, ranging from 0 (Never) to 4 (Always), indicating their agreement with the items, e.g., “*I feel that I have given up.*” Cronbach’s α for this scale was high at 0.95.

##### Entrapment

Entrapment was measured using the Entrapment Scale (Gilbert and Allan, 1998), which evaluates perceptions of being unable to escape from one’s current situation or circumstances. Sixteen self-report items, e.g., “*I have a strong desire to escape from things in my life,”* are rated on a 5-point scale from 0 (never) to 4 (always), reflecting how frequently they have been experienced. The scale showed high internal consistency at α = 0.94.

##### Coping

Coping was assessed using the Brief COPE (Carver, 1997), measuring the degree to which a person uses a specific strategy to deal with difficult or stressful situations. The 28-item measure covers various strategies, e.g., “*I have been using alcohol or other drugs to make myself feel better*,” which are evaluated using a 4-point scale from 1 (I haven’t been doing this at all) to 4 (I’ve been doing this a lot). Several scoring methods have been proposed. As per Moore et al. (2011) and Blomgren et al. (2016), we differentiated between adaptive coping (16 items covering active coping, planning, positive reframing, humour, acceptance, religion, use of emotional support, and use of instrumental support) and maladaptive coping (12 items on self-distraction, denial, substance use, behavioural disengagement, venting, and self-blame). Cronbach’s α was high for both the adaptive and maladaptive subscales, at 0.83 and 0.74, respectively.

##### Self-esteem

The Rosenberg Self-Esteem Scale (Rosenberg, 1965) was used to measure self-esteem by asking about self-worth and positive or negative feelings about oneself. Ten items are answered on a 4-point scale from 0 (Strongly Agree) to 3 (Strongly Disagree), with higher scores indicating greater self-esteem, e.g., “*I take a positive attitude toward myself.*” Internal consistency was high at α = 0.91.

##### Social support

The Multidimensional Scale of Perceived Social Support (MSPSS; Zimet et al., 1988) was used to assess the perceived adequacy of social support that an individual receives from family, friends, and significant others. Each of the three categories is assessed with four items, given on a 7-point scale ranging from 1 (very strongly disagree) to 7 (very strongly agree), e.g., “*I can talk about my problems with my family.*” A total overall score or three subscale scores for the different sources of support can be calculated by summing the relevant items. Subscales were used here to differentiate the influence of different sources of support; internal consistency was high (family α = 0.88, friends α = 0.90, and significant others α = 0.88).

##### Stigma

Attitudes toward people who die by suicide were measured using the Short Form of the Stigma of Suicide Scale (SOSS; Batterham et al., 2013b), which asks participants to rate how much they agree or disagree with words describing people who take their own lives. Sixteen items, e.g., “*irresponsible*,” “*lonely*,” “*noble*,” are rated on a 5-point scale from Strongly Disagree to Strongly Agree. Subscales of stigma, isolation/depression, and glorification/normalisation can be calculated by summing the relevant items for each subscale; Internal consistency for the respective subscales was α = 0.83, α = 0.85, and α = 0.75.

#### Volitional Phase Variables

##### Bereavement

In order to inquire about adverse life events, including bereavements, that may have occurred in young people’s lives, a 20-item Life Events Checklist (LEC) from the CASE Study Lifestyle and Coping Questionnaire (Hawton et al., 2006) was utilised. This measure asks about potentially traumatic life events occurring within the past 12 months and/or more than a year ago, and has been used in several countries with adolescent samples (e.g., Madge et al., 2011; Hasking et al., 2013). Although participants were presented with all 20-items, only five are relevant and discussed here; three items inquired about experiences with the death of someone close. Specifically, “*Has anyone among your immediate family* (*mother, father, brother, or sister*) *died?*,” “*Has anyone close to you died?*”, and “*Has anyone among your family or friends committed suicide?*” Although we acknowledged the outdated language used in this question, wording of items was left as in the original measure to allow for comparisons with the extant literature.

##### Self-harm of family or friends

Two additional questions from the Life Events Checklist inquired about self-harm behaviours among one’s friends or family: “*Has anyone among your [family]/[close friends] attempted suicide or deliberately harmed themselves?*” As with all LEC items, possible responses included ‘Yes, in the past 12 months,’ ‘Yes, more than a year ago,’ or ‘No.’

### Procedure

Ethical approval was granted from the University of Strathclyde Ethics Committee, and approval was given from 14 local education authorities across Scotland to carry out the study in their area. 153 secondary schools in participating areas were contacted and invited to take part. An information sheet detailing the nature of the investigation and consent form were sent out to all parents/guardians of pupils in participating year groups. Parental/guardian consent as well as participant consent was obtained.

The researcher then visited each school and spoke to pupils to explain the procedures and address any questions in person. Pupils were given information sheets to take away with them and read in their own time, which explained that the research would ask about past experiences of bereavement as well as self-harm or suicide. Approximately 2 weeks later, pupils were invited to complete the questionnaire during a class period. Two versions of the anonymous questionnaire with counterbalanced measures were distributed so as to avoid order effects, and ensure that respondents could not gauge the responses of their peers in answering sensitive questionnaire items and thus maintain a level of confidentiality. Participants completed them individually after providing informed consent. Completion of the questionnaire took approximately 30 min, and the researcher was present throughout to address any questions or issues arising. Pupils were provided with blank sealable envelopes to return their completed forms at the end, and were debriefed and given an information pamphlet with follow-up sources of support should they feel they need it, which was tailored to each school. Approximately 6 months later, pupils were invited to complete the questionnaire for a second time under the same conditions, and informed consent was obtained again.

### Data Analytic Plan

Missing data was dealt with using multiple imputation, as Little’s MCAR test was non-significant, χ^2^(5) = 5.09, *p* = 0.405, and data was deemed to be most likely missing completely at random. A total of *m* = 68 imputations were generated based on 68% of cases having incomplete data (as suggested by White et al., 2010). Analyses were conducted using SPSS Version 27, which supports pooled analyses based on imputed datasets for several statistical tests; however, some analyses are not supported by this function. In such cases, parameter estimates were manually averaged across the 68 imputed datasets, an approach also taken by Jones et al. (2014) when dealing with imputed data in SPSS. Microsoft Excel 2013 was used to manually pool parameter estimates where necessary.

Prevalence rates of SSHTBs, as well as bereavement experiences, were reported through descriptive statistics. Hierarchical multinomial logistic regressions were used to investigate which variables were associated with self-harm outcomes at baseline and at follow-up. Two separate regressions for each time point (baseline and follow-up) were conducted in order to test the motivational and volitional phases of the model. All analyses controlled for age and gender, given the established differences with respect to self-harm (Hawton et al., 2012). We also controlled for baseline mood, in line with previous research (e.g., O’Connor et al., 2012; Hasking et al., 2013; Dhingra et al., 2015) and given that depression and anxiety were both significantly associated with self-harm group status at T1 and T2. Odds ratios and confidence intervals were obtained from univariate analyses. Variables which were significantly associated with self-harm group status in univariate analyses were entered into multivariate analyses to determine their relative contributions. Holm–Bonferroni corrections were applied to correct for multiple comparisons.

## Results

### Prevalence of Bereavement and Self-Harm at Baseline (T1)

A comparison of those who took part at baseline only and those who participated at both time points revealed no significant differences on most of the demographic or studied variables, apart from SES, family social support, and lifetime self-harm (those followed-up came from schools with a lower proportion of pupils receiving free school meals, reported lower family social support, and were more likely to report self-harm at baseline). Descriptive statistics of continuous study variables for all participants across all self-harm groups are shown in Table 1. At baseline (*n* = 185), 136 (73.51%) young people reported that someone among their immediate family and/or someone else close had died; 21 (11.35%) of which knew someone who had died by suicide (making up the suicide exposed group), while the remaining 115 (62.16%) people were exposed to a non-suicide death.

**TABLE 1:** Descriptive statistics for continuous scale variables for participants at both time points, within each self-harm group.

	Total (*M, SD*)		Control (*M, SD*)		Ideation (*M, SD*)		Enactment (*M, SD*)	
				
	T1 (*n* = 185)	T2 (*n* = 115)	T1 (*n* = 103)	T2 (*n* = 56)	T1 (*n* = 44)	T2 (*n* = 26)	T1 (*n* = 38)	T2 (*n* = 33)
Age	13.16 (1.49)	13.65 (1.52)	12.89 (1.42)	13.30 (1.40)	13.43 (1.49)	14.08 (1.67)	13.58 (1.57)	13.91 (1.49)
SES	14.29 (5.19)	13.29 (5.22)	14.96 (4.77)	13.50 (5.10)	12.24 (5.80)	12.41 (5.50)	14.83 (5.05)	13.60 (5.26)
Depression	7.72 (7.18)	8.57 (7.82)	4.16 (4.75)	3.03 (3.36)	9.71 (6.41)	10.00 (5.49)	15.07 (7.12)	16.85 (7.00)
Anxiety	6.78 (6.26)	8.08 (7.10)	3.91 (4.83)	3.41 (4.38)	9.41 (5.85)	10.73 (6.41)	11.48 (6.11)	13.91 (5.96)
Defeat	17.90 (15.07)	20.19 (16.72)	10.50 (8.87)	9.65 (8.85)	21.96 (14.30)	21.46 (10.01)	33.26 (16.11)	37.07 (17.11)
Entrapment	14.13 (14.68)	16.31 (16.67)	6.79 (8.57)	4.98 (6.59)	18.44 (13.55)	21.75 (13.64)	29.01 (16.01)	31.26 (17.03)
Adaptive Coping	33.91 (8.79)	34.14 (9.13)	32.43 (8.56)	32.41 (10.21)	35.35 (9.52)	37.75 (6.95)	36.29 (7.89)	34.25 (7.99)
Maladaptive Coping	21.96 (5.80)	22.08 (6.84)	19.13 (4.61)	17.97 (4.39)	24.31 (4.97)	24.79 (5.42)	26.93 (5.07)	26.93 (7.14)
Self-Esteem	21.49 (6.10)	22.07 (6.50)	18.77 (4.65)	17.82 (4.36)	23.56 (6.11)	23.77 (3.54)	26.48 (5.55)	27.92 (6.24)
SS - Family	5.67 (1.44)	5.51 (1.63)	6.16 (1.08)	6.31 (0.82)	5.37 (1.32)	5.53 (1.32)	4.71 (1.81)	4.14 (1.97)
SS - Friends	5.24 (1.56)	5.31 (1.68)	5.45 (1.36)	5.40 (1.63)	5.04 (1.76)	5.56 (1.59)	4.92 (1.76)	4.96 (1.82)
SS - Significant Other	5.56 (1.50)	5.70 (1.48)	5.79 (1.30)	5.86 (1.3)	5.57 (1.48)	5.98 (1.41)	4.92 (1.86)	5.22 (1.72)
SOSS - Stigma	2.10 (0.74)	1.92 (0.74)	2.15 (0.72)	2.03 (0.76)	2.13 (0.75)	2.19 (0.81)	1.91 (0.74)	1.52 (0.48)
SOSS - Iso/Dep	3.57 (1.04)	3.71 (0.98)	3.38 (1.06)	3.31 (1.03)	3.71 (0.91)	4.17 (0.85)	3.90 (1.01)	4.03 (0.70)
SOSS - Glo/Nor	2.52 (0.90)	2.61 (0.87)	2.48 (0.93)	2.58 (0.93)	2.73 (0.94)	2.41 (0.90)	2.40 (0.77)	4.03 (0.70)

*SES, socioeconomic status; SS, social support; SOSS, Stigma of Suicide Scale; Iso/Dep, isolation/depression subscale; Glo/Nor, glorification/normalisation subscale.*

38 (20.54%) pupils reported having ever engaged in self-harm behaviours during their lifetime (enactment group), while a further 44 (23.78%) reported past self-harm ideation with no history of behaviours (ideation group). Thus, the control group at baseline consisted of 103 (55.68%) individuals with no history of self-harm or suicidal thoughts or behaviours.

### Prevalence of Bereavement and Self-Harm at Follow-Up (T2)

At follow-up, 81 participants of the T2 sample of *n* = 115 reported that someone among their immediate family and/or someone else close had died. Seventeen (14.78%) individuals overall reported knowing someone who had died by suicide (suicide exposed group), of which 10 (8.70%) were reported for the first time since T1. A further 66 (57.39%) individuals were exposed to a non-suicide death, with 13 (11.30%) reported for the first time since baseline. It is worth noting that two individuals responded ‘no’ to the death of an immediate family member or anyone close, but ‘yes’ to experiencing a suicide death of family or friends.

At follow-up, 33 (28.70%) adolescents reported ever engaging in self-harm, with 9 (7.83%) of these for the first time between Time 1 and Time 2. A further 26 (22.61%) individuals reported having experienced self-harm ideation (with no actions) at follow-up. The control group at T2 therefore comprised of 56 (58.70%) individuals who reported no history of self-harm ideation or behaviours at follow-up.

### Cross-Sectional Associations Between Motivational and Volitional Phase Variables and Self-Harm at Baseline (T1)

#### Motivational Phase Variables

A hierarchical multinomial logistic regression was conducted to examine whether motivational phase variables were associated with self-harm group status at baseline. In univariate analyses, those in the ideation group reported higher levels of entrapment (OR = 1.06, 95% CI = 1.01–1.11, *p* = 0.016), were more likely to employ maladaptive coping strategies (OR = 1.16, 95% CI = 1.04–1.30, *p* = 0.008), and report less available social support from family members (OR = 0.68, 95% CI = 0.50–0.93, *p* = 0.017) compared to controls, as expected (Table 2). Comparisons between the enactment group and controls showed similar patterns on the same variables as well as defeat (defeat: OR = 1.10, 95% CI = 1.04–1.18, *p* = 0.002; entrapment: OR = 1.09, 95% CI = 1.04–1.15, *p* = 0.001; maladaptive coping: OR = 1.22, 95% CI = 1.07–1.40, *p* = 0.003; family social support: OR = 0.57, 95% CI = 0.40–0.81, *p* = 0.002). As predicted, the ideation group did not differ from the enactment group on any motivational phase variable, apart from the enactment group being less likely to endorse glorifying/normalising beliefs about suicide (OR = 0.46, 95% CI = 0.25–0.84, *p* = 0.012).

**TABLE 2:** Univariate multinomial logistic regression of the association between motivational phase variables and self-harm group status at baseline (controlling for age, gender, depression, and anxiety).

Motivational phase variable		*B*	*SE*	OR	95% CI for Odds Ratio	*p*
**Defeat**						
Control	Ideation	0.06	0.03	1.06	1.00–1.12	0.049
Control	Enactment	0.10	0.03	1.10	1.04–1.18	**0.002**
Ideation	Enactment	0.04	0.03	1.04	0.99–1.10	0.117
**Entrapment**						
Control	Ideation	0.06	0.02	1.06	1.01–1.11	**0.016**
Control	Enactment	0.09	0.03	1.09	1.04–1.15	**0.001**
Ideation	Enactment	0.03	0.02	1.03	0.99–1.07	0.189
**Adaptive coping**						
Control	Ideation	0.02	0.02	1.02	0.97–1.07	0.411
Control	Enactment	0.04	0.03	1.04	0.98–1.11	0.157
Ideation	Enactment	0.02	0.03	1.02	0.97–1.08	0.426
**Maladaptive coping**						
Control	Ideation	0.15	0.06	1.16	1.04–1.30	**0.008**
Control	Enactment	0.20	0.07	1.22	1.07–1.40	**0.003**
Ideation	Enactment	0.05	0.06	1.05	0.94–1.18	0.396
**Self-esteem**						
Control	Ideation	0.11	0.05	1.12	1.01–1.24	0.035
Control	Enactment	0.12	0.06	1.12	0.99–1.27	0.066
Ideation	Enactment	0.00	0.06	1.00	0.90–1.12	0.970
**SS - family**						
Control	Ideation	–0.39	0.16	0.68	0.50–0.93	**0.017**
Control	Enactment	–0.57	0.18	0.57	0.40–0.81	**0.002**
Ideation	Enactment	–0.19	0.16	0.83	0.61–1.13	0.229
**SS - friends**						
Control	Ideation	–0.07	0.13	0.93	0.72–1.22	0.608
Control	Enactment	–0.03	0.16	0.97	0.71–1.33	0.850
Ideation	Enactment	0.04	0.15	1.04	0.78–1.39	0.793
**SS - significant other**						
Control	Ideation	–0.11	0.15	0.89	0.67–1.19	0.431
Control	Enactment	–0.28	0.17	0.75	0.54–1.04	0.089
Ideation	Enactment	–0.17	0.16	0.84	0.62–1.16	0.292
**SOSS - stigmatisation**						
Control	Ideation	–0.17	0.30	0.84	0.47–1.53	0.575
Control	Enactment	–0.65	0.38	0.52	0.25–1.09	0.084
Ideation	Enactment	–0.48	0.36	0.62	0.30–1.26	0.185
**SOSS - Iso/Dep**						
Control	Ideation	–0.06	0.23	0.95	0.60–1.50	0.813
Control	Enactment	–0.03	0.31	0.97	0.53–1.80	0.928
Ideation	Enactment	0.03	0.31	1.03	0.56–1.88	0.931
**SOSS - Glo/Nor**						
Control	Ideation	0.20	0.24	1.22	0.75–1.96	0.423
Control	Enactment	–0.59	0.32	0.55	0.30–1.03	0.060
Ideation	Enactment	–0.79	0.32	0.46	0.25–0.84	**0.012**

*Holm-Bonferroni corrections were applied; only the comparisons in bold remain significant at the adjusted significance level. SES, socioeconomic status. SS, social support; SOSS, Stigma of Suicide Scale; Iso/Dep, isolation/depression subscale; Glo/Nor, glorification/normalisation subscale.*

Significant univariate predictors associated with self-harm were entered into a multivariate analysis (Table 3), which found that three factors continued to be associated with self-harm group membership: ideation (OR = 1.15, 95% CI = 1.02–1.30, *p* = 0.022) and enactment (OR = 1.22, 95% CI = 1.04–1.42, *p* = 0.012) groups weremore likely to report maladaptive coping compared to controls, as predicted; ideation (OR = 0.67, 95% CI = 0.47–0.94, *p* = 0.020) and enactment (OR = 0.60, 95% CI = 0.39–0.91, *p* = 0.016) groups were both more likely to report lower family social support compared to controls as predicted, and the enactment group were also less likely to hold glorifying/normalising beliefs about suicide than the ideation group (OR = 0.42, 95% CI = 0.22–0.80, *p* = 0.009).

**TABLE 3:** Multivariate multinomial logistic regression of the association between motivational phase variables and self-harm group status at baseline (controlling for age, gender, depression, and anxiety).

Motivational phase variable		*B*	*SE*	OR	95% CI for Odds Ratio	*p*
**Defeat**						
Control	Ideation	0.03	0.03	1.04	0.97–1.10	0.273
Control	Enactment	0.07	0.04	1.07	1.00–1.15	0.061
Ideation	Enactment	0.04	0.03	1.04	0.97–1.10	0.260
**Entrapment**						
Control	Ideation	0.03	0.03	1.03	0.97–1.08	0.336
Control	Enactment	0.04	0.03	1.04	0.97–1.10	0.281
Ideation	Enactment	0.01	0.03	1.01	0.96–1.07	0.764
**Maladaptive coping**						
Control	Ideation	0.14	0.06	1.15	1.02–1.30	**0.022**
Control	Enactment	0.20	0.08	1.22	1.04–1.42	**0.012**
Ideation	Enactment	0.05	0.07	1.05	0.92–1.21	0.450
**SS - family**						
Control	Ideation	–0.41	0.17	0.67	0.47–0.94	**0.020**
Control	Enactment	–0.51	0.21	0.60	0.39–0.91	**0.016**
Ideation	Enactment	–0.11	0.18	0.90	0.63–1.28	0.554
**SOSS - Glo/Nor**						
Control	Ideation	0.22	0.26	1.25	0.75–2.08	0.397
Control	Enactment	–0.66	0.35	0.52	0.26–1.04	0.062
Ideation	Enactment	–0.88	0.34	0.42	0.22–0.80	**0.009**

*Holm-Bonferroni corrections were applied; only the comparisons in bold remain significant at the adjusted significance level. SES, socioeconomic status; SS, social support; SOSS, Stigma of Suicide Scale; Glo/Nor, glorification/normalisation subscale.*

#### Volitional Phase Variables

A similar logistic regression analysis was conducted to examine volitional phase variables and their association with self-harm group status at baseline. Univariate analyses showed that ideation and enactment groups did not differ from controls on any variable. The ideation group differed from the enactment group only on family self-harm (OR = 0.17, 95% CI = 0.04–0.70, *p* = 0.014), where those who self-harmed were more likely to report this experience (Table 4). Neither experiencing a suicide nor a non-suicide death were associated with self-harm group membership. A multivariate analysis was not conducted as only one variable emerged as a significant predictor in this analysis.

**TABLE 4:** Univariate multinomial logistic regression of the association between volitional phase variables and self-harm group status at baseline (controlling for age, gender, depression, and anxiety).

Volitional phase variable		*B*	*SE*	OR	95% CI for Odds Ratio	*p*
**Suicide death**						
Control	Ideation	0.02	0.80	1.02	0.21–4.91	0.977
Control	Enactment	–1.18	0.74	0.31	0.07–1.31	0.111
Ideation	Enactment	–1.20	0.68	0.30	0.08–1.13	0.075
**Non-suicide death**						
Control	Ideation	0.14	0.44	1.15	0.49–2.71	0.744
Control	Enactment	0.32	0.51	1.37	0.50–3.74	0.538
Ideation	Enactment	0.17	0.50	1.19	0.45–3.16	0.729
**Family self-harm**						
Control	Ideation	1.16	0.79	3.20	0.69–14.93	0.139
Control	Enactment	–0.61	0.64	0.54	0.16–1.90	0.341
Ideation	Enactment	–1.77	0.72	0.17	0.04–0.70	**0.014**
**Friend self-harm**						
Control	Ideation	–0.67	0.45	0.51	0.21–1.24	0.137
Control	Enactment	–0.93	0.52	0.39	0.14–1.08	0.071
Ideation	Enactment	–0.27	0.50	0.77	0.29–2.03	0.594

*Holm-Bonferroni corrections were applied; only the comparisons in bold remain significant at the adjusted significance level.*

### Longitudinal Associations Between Motivational and Volitional Phase Variables and Self-Harm at Follow-Up (T2)

#### Motivational Phase Variables

A hierarchical multinomial logistic regression examined whether motivational phase variables were associated with life-time self-harm group 6-months later. In univariate analyses, participants in the ideation group were significantly more likely than controls to have reported self-harm ideation at baseline (Table 5a; OR = 0.08, 95% CI = 0.02–0.34, *p* = 0.001). Those in the enactment group differed from controls on family social support (OR = 0.53, 95% CI = 0.33–0.87, *p* = 0.011), stigmatising beliefs about suicide (OR = 0.27, 95% CI = 0.09–0.78, *p* = 0.016), glorifying/normalising beliefs about suicide (OR = 0.31, 95% CI = 0.12–0.76, *p* = 0.010), and self-harm ideation at baseline (OR = 0.05, 95% CI = 0.01–0.26, *p* = 0.001). The enactment group did not differ from the ideation group on any motivational phase variable.

**TABLE 5a:** Univariate multinomial logistic regression of the association between motivational phase variables and self-harm group status at follow-up (controlling for age, gender, depression, and anxiety).

Motivational phase variable		*B*	*SE*	OR	95% CI for Odds Ratio	*p*
**Defeat**						
Control	Ideation	0.03	0.04	1.03	0.96–1.10	0.475
Control	Enactment	0.02	0.04	1.02	0.94–1.10	0.696
Ideation	Enactment	–0.01	0.03	0.99	0.93–1.06	0.757
**Entrapment**						
Control	Ideation	0.07	0.04	1.07	1.00–1.14	0.053
Control	Enactment	0.07	0.04	1.07	1.00–1.16	0.055
Ideation	Enactment	0.01	0.03	1.01	0.95–1.06	0.873
**Adaptive coping**						
Control	Ideation	0.00	0.04	1.00	0.94–1.08	0.923
Control	Enactment	–0.07	0.04	0.93	0.86–1.01	0.091
Ideation	Enactment	–0.08	0.04	0.93	0.86–1.01	0.066
**Maladaptive coping**						
Control	Ideation	0.04	0.08	1.04	0.89–1.22	0.611
Control	Enactment	–0.01	0.09	1.00	0.84–1.18	0.952
Ideation	Enactment	–0.05	0.08	0.96	0.82–1.11	0.559
**Self-esteem**						
Control	Ideation	0.05	0.07	1.05	0.91–1.21	0.514
Control	Enactment	0.08	0.08	1.08	0.92–1.28	0.348
Ideation	Enactment	0.03	0.08	1.03	0.89–1.20	0.688
**SS - family**						
Control	Ideation	–0.17	0.23	0.85	0.54–1.32	0.466
Control	Enactment	–0.63	0.25	0.53	0.33–0.87	**0.011**
Ideation	Enactment	–0.47	0.22	0.63	0.41–0.96	0.034
**SS - friends**						
Control	Ideation	–0.01	0.19	0.99	0.69–1.44	0.972
Control	Enactment	0.09	0.23	1.10	0.70–1.71	0.687
Ideation	Enactment	0.10	0.20	1.10	0.74–1.64	0.628
**SS - significant other**						
Control	Ideation	–0.25	0.20	0.78	0.53–1.17	0.231
Control	Enactment	–0.52	0.22	0.60	0.39–0.93	0.022
Ideation	Enactment	–0.27	0.20	0.76	0.51–1.14	0.186
**SOSS - stigmatisation**						
Control	Ideation	–0.28	0.44	0.76	0.32–1.80	0.533
Control	Enactment	–1.32	0.55	0.27	0.09–0.78	**0.016**
Ideation	Enactment	–1.05	0.51	0.35	0.13–0.96	0.041
**SOSS - Iso/Dep**						
Control	Ideation	0.09	0.35	1.09	0.55–2.18	0.808
Control	Enactment	–0.14	0.41	0.87	0.39–1.95	0.741
Ideation	Enactment	–0.22	0.40	0.80	0.37–1.76	0.582
**SOSS - Glo/Nor**						
Control	Ideation	–0.55	0.38	0.58	0.27–1.21	0.145
Control	Enactment	–1.18	0.46	0.31	0.12–0.76	**0.010**
Ideation	Enactment	–0.63	0.40	0.53	0.24–1.17	0.118
**Self-harm ideation at T1**						
Control	Ideation	–2.51	0.73	0.08	0.02–0.34	**0.001**
Control	Enactment	–3.08	0.89	0.05	0.01–0.26	**0.001**
Ideation	Enactment	–0.56	0.83	0.57	0.11–2.90	0.498

*Holm-Bonferroni corrections were applied; only the comparisons in bold remain significant at the adjusted significance level. SES, socioeconomic status; SS, social support; SOSS, Stigma of Suicide Scale; Iso/Dep, isolation/depression subscale; Glo/Nor, glorification/normalisation subscale.*

Significant univariate predictors associated with self-harm were entered into a multivariate analysis (Table 5b), which found that three factors continued to be associated with self-harm group membership: the ideation group were less likely to report glorifying/normalising beliefs about suicide (OR = 0.19, 95% CI = 0.06–0.66, *p* = 0.009), and more likely to report self-harm ideation at baseline (OR = 0.03, 95% CI = 0.00–0.20, *p* < 0.001) compared to controls. In addition, the enactment group were less likely to hold stigmatising beliefs about suicide (OR = 0.18, 95% CI = 0.05–0.73, *p* = 0.016), less likely to hold glorifying/normalising beliefs about suicide (OR = 0.11, 95% CI = 0.03–0.46, *p* = 0.002), and more likely to have reported self-harm ideation at baseline (OR = 0.02, 95% CI = 0.00–0.22, *p* = 0.002), compared to controls.

**TABLE 5b:** Multivariate multinomial logistic regression of the association between motivational phase variables and self-harm group status at follow-up (controlling for age, gender, depression, and anxiety).

Motivational phase variable		*B*	*SE*	OR	95% CI for Odds Ratio	*p*
**SS - family**						
Control	Ideation	–0.09	0.28	0.91	0.53–1.59	0.747
Control	Enactment	–0.57	0.31	0.57	0.31–1.04	0.067
Ideation	Enactment	–0.47	0.23	0.62	0.39–0.99	0.043
**SOSS - stigmatisation**						
Control	Ideation	–0.78	0.57	0.46	0.15–1.41	0.173
Control	Enactment	–1.70	0.70	0.18	0.05–0.73	**0.016**
Ideation	Enactment	–0.92	0.55	0.40	0.14–1.17	0.094
**SOSS - Glo/Nor**						
Control	Ideation	–1.64	0.63	0.19	0.06–0.66	**0.009**
Control	Enactment	–2.20	0.72	0.11	0.03–0.46	**0.002**
Ideation	Enactment	–0.56	0.46	0.57	0.23–1.42	0.230
**Self-harm ideation at T1**						
Control	Ideation	–3.54	0.98	0.03	0.00–0.20	<**0.001**
Control	Enactment	–3.97	1.26	0.02	0.00–0.22	**0.002**
Ideation	Enactment	–0.43	0.99	0.65	0.09–4.56	0.668

*Holm-Bonferroni corrections were applied; only the comparisons in bold remain significant at the adjusted significance level. SES, socioeconomic status; SS, social support; SOSS, Stigma of Suicide Scale; Iso/Dep, isolation/depression subscale; Glo/Nor, glorification/normalisation subscale.*

#### Volitional Phase Variables

Another analysis was conducted to examine volitional phase variables and their association with self-harm group status prospectively. In univariate tests, none of the variables, including experiencing a suicide or a non-suicide death, emerged as significant predictors of self-harm group membership (Table 6). A multivariate analysis was therefore not required.

**TABLE 6:** Univariate multinomial logistic regression of the association between volitional phase variables and self-harm group status at follow-up (controlling for age, gender, depression, and anxiety).

Volitional phase variable		*B*	*SE*	OR	95% CI for Odds Ratio	*p*
**Suicide death**						
Control	Ideation	–2.02	1.41	0.13	0.01–2.12	0.153
Control	Enactment	–2.31	1.45	0.10	0.01–1.72	0.112
Ideation	Enactment	–0.29	0.82	0.75	0.15–3.74	0.725
**Non-suicide death**						
Control	Ideation	–0.30	0.64	0.74	0.21–2.57	0.635
Control	Enactment	–1.01	0.79	0.37	0.08–1.72	0.202
Ideation	Enactment	–0.71	0.74	0.49	0.12–2.08	0.336
**Family self-harm**						
Control	Ideation	–0.70	0.95	0.50	0.08–3.19	0.462
Control	Enactment	–1.44	0.96	0.24	0.04–1.55	0.134
Ideation	Enactment	–0.74	0.79	0.48	0.10–2.24	0.347
**Friend self-harm**						
Control	Ideation	0.18	0.65	1.20	0.34–4.25	0.776
Control	Enactment	–1.17	0.70	0.31	0.08–1.22	0.094
Ideation	Enactment	–1.35	0.67	0.26	0.07–0.97	0.044

*Holm-Bonferroni corrections were applied; only the comparisons in bold remain significant at the adjusted significance level.*

Overall, cross-sectional analyses showed that maladaptive coping, family social support and endorsing glorifying/normalising beliefs about suicide (motivational phase variables) and family self-harm (volitional phase variable) were significant predictors of self-harm group status. Longitudinally, endorsing stigmatising beliefs about suicide, endorsing glorifying/normalising beliefs about suicide, and self-harm ideation at baseline (motivational phase variables) predicted self-harm group at follow-up.

## Discussion

This study aimed to investigate whether experiences of bereavement and other theoretically derived variables were associated with self-harm group status. These relationships were examined both cross-sectionally and over a 6-month period, given that longitudinal work is crucial for establishing causal relationships and constitutes particularly strong evidence. As suggested by the IMV model, participants in the ideation and enactment groups were expected to differ from controls, but not each other, on motivational phase variables, namely: defeat, entrapment, social support, coping, self-esteem, and attitudes to suicide. In addition, as predicted by the volitional phase of the model, it was expected that the ideation and enactment groups would differ from controls and each other on exposure to suicide deaths, family self-harm, and friend self-harm, but not exposure to non-suicide deaths.

Results partially supported the hypotheses both cross-sectionally and longitudinally. Although several variables (defeat, entrapment, maladaptive coping, family social support, and endorsing glorifying/normalising beliefs about suicide) predicted self-harm group membership in univariate analyses, only maladaptive coping, social support from family members and endorsing glorifying/normalising beliefs about suicide remained significant multivariate predictors within the motivational phase of the model. Family self-harm was the only predictor among the volitional phase variables to predict self-harm group cross-sectionally. Results of longitudinal analyses showed that endorsing stigmatising beliefs about suicide, endorsing glorifying/normalising beliefs about suicide, and self-harm ideation (motivational phase test) at baseline predicted self-harm group membership 6-months later, with none of the volitional phase variables emerging as significant predictors of prospective self-harm.

### Bereavement Experiences as Predictors of Self-Harm

Contrary to model predictions, bereavement did not predict self-harm group at either time point. Wetherall et al. (2018) reported on cross-sectional data from the Scottish Wellbeing Study of 18–34 year olds, and found that having a friend who attempted suicide differentiated ideation from enactment groups as expected, but having a family member or friend die by suicide did not. This study was based on a comparatively large sample (suicide attempt *n* = 403, suicidal ideation *n* = 498, control *n* = 2,534) and similarly did not find evidence to support an effect of loss to suicide. Future investigations should examine other features surrounding the death, such as the time elapsed since the death, closeness/quality of the relationship, or mental health history (Pitman et al., 2014; Andriessen et al., 2016).

It is worth noting that the rates of suicide and non-suicide death exposure reported in this sample reflect rates from previous research with young people. Harrison and Harrington (2001) reported that 77.6% of 11–16 year olds were bereaved of a relative or close friend. Madge et al. (2011), using data from the CASE Study which utilised the LEC measure, found that 59.7% of adolescents had experienced the death of someone close, and 30.4% had experienced a suicide death *or* self-harm of others (the authors did not differentiate fatal from non-fatal self-harm). However, it is worth noting that the LEC measure may not be reflective of the number of adolescents who consider themselves suicide bereaved. Indeed, two individuals at T2 reported no deaths of immediate family members or other close persons, but simultaneously reported a death to suicide of a family member or friend at the same time point. Based on the information gathered in this study, it is unclear whether this was due to the wording of the question, recall bias, or whether those reporting a family member or friend who died by suicide would consider themselves *bereaved by* suicide or *exposed to* suicide, given that follow-up information could not be ascertained regarding the extent to which they were impacted. This relates to the issue of terminology discussed by Cerel et al. (2014), who propose a continuum of suicide bereavement, where an individual can be exposed, affected, or bereaved (short/long-term) by suicide; these categories reflect varying levels of emotional attachment and adjustment after the loss. It has been said that simply being exposed to a suicide death does not constitute someone being deeply affected by the death (Andriessen et al., 2017). While limited conclusions can be made on this based on the data collected, it is nonetheless apparent that a large proportion of adolescents reported a suicide death of someone they knew.

### IMV Model Psychological Variables as Predictors of Self-Harm

As predicted, maladaptive coping was found to be associated with self-harm group at baseline, and both ideation and enactment group participants differed from controls but not each other. This is in keeping with the large body of research that associates low levels of coping skills with suicidal thoughts and feelings (Gooding et al., 2015). Research particularly highlights that avoidant or emotion-focused (rather than problem-focused) coping styles are associated with self-harm in adolescents (Guerreiro et al., 2013), including the use of alcohol and drugs, behavioural disengagement and self-blame strategies, which were measured by the maladaptive coping subscale used in this study. The finding that only the maladaptive subscale was significant may reflect that self-harm might represent a coping style in itself; Laye-Gindhu and Schonert-Reichl (2005) suggest that self-harm is an emotion-focused strategy that serves to regulate affect. Indeed, research on motivations for self-harm among adolescents has found that getting relief from a terrible state of mind was the strongest predictor of self-harm (Rasmussen et al., 2016), which may account for the significant association found here.

Our cross-sectional findings reflect previous research (Kleiman and Liu, 2013; O’Connor and Nock, 2014) and theory (O’Connor, 2011; O’Connor and Kirtley, 2018) showing that levels of social support are significantly associated with suicide risk. In a recent large-scale study, Wan et al. (2019) found that lower social support was significantly associated with self-reported NSSI, suicidal ideation and suicide attempts among young people aged 10–20 years old. Our finding that only family social support was associated with self-harm group membership is consistent with Cheng and Chan (2007); using a translated version of the MSPSS, they found that the impact of family social support was stronger than that of friends in predicting suicidality among adolescents. Similarly, Tabaac et al. (2016) reported that social support from family and significant others was associated with suicidal ideation, but only family social support was associated with suicide attempts. They suggest that family members may represent a closer and more permanent source of support than other social groups, particularly for adolescents dealing with stressful life events.

As predicted, endorsing stigmatising beliefs about suicide significantly differentiated controls from enactment groups in longitudinal analyses, and glorifying/normalising beliefs differentiated controls from both ideation and enactment groups prospectively. It was found that higher levels of such beliefs were associated with being in the control group. The finding of a significant association at baseline in the motivational phase variable of glorifying/normalising beliefs about suicide was contrary to IMV model predictions, as ideation and enactment groups were not expected to differ. The ideation group were more likely to endorse glorifying or normalising beliefs about suicide than the enactment group at baseline. Previous research using the same SOSS measure (Batterham et al., 2013a) also showed that suicidal ideation was associated with greater glorification of suicide, as well as less stigma toward suicide, whereas suicide attempts were not associated with any attitude subscale (stigma, isolation/depression, or glorification/normalisation). One possible explanation for both cross-sectional and longitudinal findings is that individuals who self-harm are more likely to have been exposed to similar behaviours in others (Dhingra et al., 2015; Mars et al., 2019b), and increased exposure has been shown to reduce stigma (e.g., in relation to mental disorders; Jorm and Wright, 2008); in this study, experiencing self-harm of family members was indeed associated with self-harm group status cross-sectionally, which may account for the lack of an association with glorifying/normalising beliefs among the enactment group. Interestingly, self-harm group status was not associated with suicide bereavement. Given the small numbers of young people bereaved by suicide it was beyond the scope of this research to compare different bereavement groups. However, Bartik et al. (2015) found that those bereaved by suicide were more likely than the general population to perceive suicide as stigmatising and in glorifying or normalising terms, and less likely to attribute it to isolation and depression. Future research should therefore endeavour to assess how attitudes impact help-seeking among those who are suicide bereaved, to better understand the relationship between attitudes and self-harm.

The finding that baseline self-harm ideation predicted self-harm group at follow-up is consistent with past research (Ribeiro et al., 2016) and the theoretical assertion that ideation/intention is a proximal predictor of engagement in behaviours (O’Connor, 2011; O’Connor and Kirtley, 2018). We also found that self-harm among family members could predict self-harm group membership at baseline. In a UK population-based cohort study, Mars et al. (2019a) showed that exposure to family self-harm was a predictor of future suicide attempts among adolescents who reported suicidal thoughts (but not those who engaged in NSSI). O’Connor et al. (2009a) found that adolescents who engaged in repeat self-harm over a 6-month period were also significantly more likely to have family and friends who self-harmed than those who did not report self-harm; however, only family self-harm remained a significant predictor in multivariate analyses. These findings may be explained by familial transmission of suicidal behaviour (O’Connor et al., 2009a; Pitman et al., 2014), possibly through increased risk from shared environmental stressors or genetic factors, or transmission of psychopathology and impulsive aggression (Brent et al., 2002; Melhem et al., 2007). On the other hand, the finding that self-harm of friends did not predict self-harm group status here may also be attributed to a lack of statistical power, as numerous studies have suggested a role for social modelling of self-harm among non-family members. Self-harm among peers significantly predicted future suicidal behaviour in four large-scale studies across various countries (De Leo and Heller, 2008), where the sample sizes ranged from *n* = 731 to 11,572, depending on the study time point. This effect is observed in studies specifically with adolescents (Hawton et al., 2002; Doyle et al., 2015). Given the small sample in this study, further work is required to test this using a larger dataset.

Overall, some support for the IMV model was found. That several factors did not predict self-harm group membership cross-sectionally nor longitudinally may likely be the result of limited statistical power. The baseline self-harm groups consisted of 38 people in the enactment group, 44 in the ideation group, and 103 controls. At follow up, there were only 33 individuals in the self-harm enactment group, 26 in the ideation group, and 56 controls. While the sample sizes were deemed adequate for the analyses chosen, they may not have been sufficient to detect group differences, if these existed, where cell sizes were small (e.g., one third of cells had values of less than 5 in the suicide death and family self-harm variables at T2). Risk factors for self-harm can vary significantly over time and even within a day (Kashyap et al., 2015), so estimating future outcomes from measures taken 6 months earlier is particularly challenging, especially when using a small sample. It is also possible that the IMV model does not appropriately model the relationship between certain variables, or may not be applicable to young people in a Scottish context. Given the absence of an association between various established risk factors (including defeat and entrapment) and self-harm in multivariate analyses, additional research is needed to determine whether these findings hold with a larger sample, and ultimately whether the model requires further refinement. Future research should also examine the difference between internal and external entrapment; we refrained from exploring this due to the small sample size.

### Strengths and Limitations

This study contributes important knowledge regarding rates of adolescent bereavement in Scotland, by suicide and generally, and the results show that rates are similar to previously published research. It also adds to the growing body of evidence testing the IMV model with adolescent community samples, showing the potential utility of this theoretical framework. A major strength of this study is its longitudinal design. While there is existing cross-sectional research demonstrating associations between bereavement and self-harm thoughts or behaviours, this has yet to be properly investigated prospectively, especially with adolescent community samples. Most longitudinal studies have been based on hospital records or national health registers (e.g., Kuramoto et al., 2013; Rostila et al., 2013; Li et al., 2014), which can be advantageous in their levels of accuracy and completeness, but do not generally capture community-occurring self-harm which seldom presents to clinical settings. Second, the variables of interest were all chosen on the basis of theory, and the specific measures were selected due to their suitability for use with adolescents. Measures were also counterbalanced to avoid order effects. The main analyses adjusted for demographic and mood variables, consistent with previous research and thereby reducing the chance of confounding. Schools were recruited from areas across the country with varying degrees of socioeconomic deprivation. As a result, the sample was reflective of the demographics of the wider population.

We recognise there are limitations to this study, particularly related to the small sample size and statistical power. Challenges with recruitment for various reasons (including school unease about the nature of the study, low response rates from schools or parents, and attrition over the study period) meant that study uptake was slow. The criteria for participation also excluded pupils who had been bereaved within the last 6 months, as suggested by previous research (Dyregrov et al., 2011), however, this period of particular risk may reflect specific experiences and needs that could not be captured here. Future research may also benefit from investigating a time period of greater than 6 months, to understand the longer-term effects of suicide bereavement. In addition, missing data in quantitative studies is often inevitable and a rate of 15–20% can be typical of educational and psychological research (Dong and Peng, 2013). In this case, the rate of missing data across individual items in the study was relatively low, at 6.13%, and data was deemed to be missing completely at random. Nevertheless, this may have reduced the statistical power and led to biassed parameter estimates, despite that multiple imputation was used to address missing values. Finally, although age, gender and mood were controlled for in all analyses, we cannot discount the possibility of residual confounding.

### Implications

These findings offer an important contribution to the limited literature on adolescent bereavement experiences and their relation to self-harm. Results highlight that self-harm ideation and behaviours are prevalent among Scottish youth, and a large proportion of adolescents have also been bereaved or exposed to the death of someone close to them. Given the potential consequences of bereavement, and particularly suicide bereavement with its association to adverse outcomes, understanding the extent and nature of this experience among adolescents is essential. As a test of a theoretical model, support is promising for some aspects of the IMV model, in particular identifying stigmatising and glorifying/normalising beliefs about suicide, and self-harm ideation as predictors of future behaviours. At the same time, evidence which was not wholly consistent with regards to the role of other variables within the IMV model, such as the impact of experiences of suicide loss, requires further investigation with larger samples to assess their placement within the model. In addition to guiding future research and theory refinement, our findings have implications on targets for clinical interventions and postvention. Efforts aimed at enhancing healthy coping skills, increasing family cohesion and social support, and addressing beliefs and attitudes about suicide (such as viewing suicide as stigmatised or glorified), and targeting self-harm ideation before it becomes severe, may be especially effective.

Future research is needed to establish whether these results replicate with a larger group and with other types of bereavements. Further qualitative work would also be beneficial to understand the impact of surviving a loss beyond what questionnaire-based methods can provide. Research which includes people bereaved more recently (e.g., within the first 6 months after a death) would also help understand whether these results are applicable to all young people regardless of their length of bereavement, and would help inform immediate postvention responses according to need. Taken as a whole, this study provides novel insight into the experience of bereavement among young people in Scotland, and within the context of an emerging theoretical model of suicide, offers potential avenues for effective intervention.

## Data Availability Statement

The data underpinning this research are available on request. Anonymous data will be archived and made available from the United Kingdom Data Service, and linked to the University of Strathclyde’s Research Data Repository. Some data will be removed in order to ensure participant confidentiality and privacy.

## Ethics Statement

The studies involving human participants were reviewed and approved by University of Strathclyde Ethics Committee. Informed consent to participate in this study was provided by the participants’ legal guardian/next of kin.

## Author Contributions

LC, SR, and SP contributed to the conceptualisation and design of the study. LC was involved in the acquisition of data. LC and SR contributed to the analysis of data. LC, SR, and SP were involved in the interpretation of results and writing of the manuscript.

## Conflict of Interest

The authors declare that the research was conducted in the absence of any commercial or financial relationships that could be construed as a potential conflict of interest.
