# Corrigendum: A Theory-Based Longitudinal Investigation Examining Predictors of Self-Harm in Adolescents With and Without Bereavement Experiences

**DOI:** 10.3389/fpsyg.2021.655774

**Published:** 2021-03-05

**Authors:** Laura del Carpio, Susan Rasmussen, Sally Paul

**Affiliations:** ^1^School of Psychological Sciences & Health, University of Strathclyde, Glasgow, United Kingdom; ^2^School of Social Work & Social Policy, University of Strathclyde, Glasgow, United Kingdom

**Keywords:** adolescence, self-harm, suicide, bereavement, theory, IMV model

In the original article, there was a mistake in the data matching and resulting statistics/text as published. A data processing error occurred whilst combining participant data across both time points, which led to a proportion of the baseline data being incorrectly matched to the follow-up data. Upon rectifying this error and re-running the analyses, we found that the main results and conclusions remain unaffected, such that bereavement experiences (by suicide or non-suicide causes) continue to show no association with self-harm group membership at baseline nor at follow-up. We did, however, find that the specific descriptive and inferential statistics have changed slightly throughout the paper as a result of the corrected matching. Our cross-sectional findings remain largely unaffected, although one variable (maladaptive coping) which was previously approaching significance has now reached statistical significance. Furthermore, two additional variables have emerged as significant in our longitudinal analyses (endorsing stigmatising beliefs, and glorifying/normalising beliefs about suicide predict prospective self-harm), and one variable (family self-harm) is no longer a significant predictor of prospective self-harm. The overall conclusions of the paper are not affected, and the additional predictors which have emerged do not have a major influence on the interpretation of our findings. The corrected paragraphs and tables appear below, as well as additional comments in the Results and Discussion section and the insertion of **Table 5b**, which address the changes in the results noted above.

Corrections have been made to *Abstract, Methods*, and *Results* sections, as shown below:

**[ABSTRACT]**

**Methods:** A 6-month prospective questionnaire study was conducted with 185 Scottish adolescents aged 11–17 (115 adolescents also completed the questionnaire at follow-up). The questionnaire included measures on experiences with bereavement and lifetime engagement in self-harm, as well as measures of defeat, entrapment, social support, coping, and other psychological variables.

**Results:** At baseline, 11% of young people reported exposure to a suicide death, and 62% to a non-suicide death. In addition, 21% of pupils reported ever engaging in self-harm, while 24% had experienced self-harm ideation without engaging in it. Cross-sectional multivariate logistic regressions showed that maladaptive coping, family social support, glorifying/normalising beliefs about suicide, and family self-harm were significantly associated with self-harm group membership (control, ideation, or enactment groups). At follow-up, 9% of pupils reported exposure to a suicide death and 11% to a non-suicide death for the first time. A total of 29% of the sample reported self-harm at T2 (8% of participants for the first time), and 23% reported self-harm ideation without engaging in it. Multivariate analyses found that stigmatising beliefs about suicide, glorifying/normalising beliefs about suicide, and self-harm ideation at baseline were the only variables to predict self-harm group membership prospectively. Bereavement experiences, whether by suicide or non-suicide, did not predict self-harm group status at baseline nor at follow-up.

Corrections have also been made to *Materials and Methods, Participants, Paragraphs 1-3*. The corrected text is shown below:

**[MATERIALS AND METHODS]**

**Participants**

A total of 185 pupils (aged 11–17, *M* = 13.16, *SD* = 1.49) were recruited at T1 from nine secondary schools across Scotland. This sample consisted of individuals retained after removing participants with >50% missing data (*n* = 2) or who did not provide data on any of the SSHTB outcome measures (*n* = 22), and including participants from T2 who only provided data once and not at baseline (i.e., were absent at the first time point, or baseline data was removed due to missingness but T2 questionnaire was complete; *n* = 15). Of the T1 sample, 85 stated they were male, 97 female, 2 other, and 1 did not respond. Approximately half were in Year 1 (predominantly aged 12–13) of secondary school (*n* = 91, 49.2%) and described their ethnicity as White (*n* = 164, 88.65%), consistent with the last Scottish Census (96.1%; National Records of Scotland, 2011). The percentage of pupils entitled to free school meals, as a proxy measure of Socioeconomic Status (SES), ranged from 4.74 to 20.99% between schools (*M* = 14.29, *SD* = 5.19), slightly lower than previous Scottish studies (e.g., mean of 17.8% in Russell et al., 2018), though comparable to the national average of 14.4% (Scottish Government, 2018).

One hundred and fifteen individuals (aged 12–18, *M* = 13.65, *SD* = 1.52; 46 male, 67 female, 2 other) provided data for T2, which could be matched to corresponding baseline data. This sample was retained after removing data from respondents whose T2 participant identifier codes could not be confidently matched to their baseline data (*n* = 31), who had >50% missing data (*n* = 3), or who did not respond to any of the outcome measures (*n* = 5).

The retention rate of 62.16% is similar to other longitudinal studies using adolescent samples (Boergers and Spirinto, 2003; O'Connor et al., 2009a; Hasking et al., 2013, 2015; Rasmussen et al., 2016).

Corrections have also been made to *Measures, Motivational Phase Variables*, sections *Entrapment, Coping, Self-Esteem, Social Support*, and *Stigma*. The corrected sections are shown below:

***Entrapment***

Entrapment was measured using the Entrapment Scale (Gilbert and Allan, 1998), which evaluates perceptions of being unable to escape from one's current situation or circumstances. Sixteen self-report items, e.g., “*I have a strong desire to escape from things in my life,”* are rated on a 5-point scale from 0 (never) to 4 (always), reflecting how frequently they have been experienced. The scale showed high internal consistency at α = 0.94.

***Coping***

Coping was assessed using the Brief COPE (Carver, 1997), measuring the degree to which a person uses a specific strategy to deal with difficult or stressful situations. The 28-item measure covers various strategies, e.g., “*I have been using alcohol or other drugs to make myself feel better*,” which are evaluated using a 4-point scale from 1 (I haven't been doing this at all) to 4 (I've been doing this a lot). Several scoring methods have been proposed. As per Moore et al. (2011) and Blomgren et al. (2016), we differentiated between adaptive coping (16 items covering active coping, planning, positive reframing, humour, acceptance, religion, use of emotional support, and use of instrumental support) and maladaptive coping (12 items on self-distraction, denial, substance use, behavioural disengagement, venting, and self-blame). Cronbach's α was high for both the adaptive and maladaptive subscales, at 0.83 and 0.74, respectively.

***Self-esteem***

The Rosenberg Self-Esteem Scale (Rosenberg, 1965) was used to measure self-esteem by asking about self-worth and positive or negative feelings about oneself. Ten items are answered on a 4-point scale from 0 (Strongly Agree) to 3 (Strongly Disagree), with higher scores indicating greater self-esteem, e.g., “*I take a positive attitude toward myself*.” Internal consistency was high at α = 0.91.

***Social support***

The Multidimensional Scale of Perceived Social Support (MSPSS; Zimet et al., 1988) was used to assess the perceived adequacy of social support that an individual receives from family, friends, and significant others. Each of the three categories is assessed with four items, given on a 7-point scale ranging from 1 (very strongly disagree) to 7 (very strongly agree), e.g., “*I can talk about my problems with my family*.” A total overall score or three subscale scores for the different sources of support can be calculated by summing the relevant items. Subscales were used here to differentiate the influence of different sources of support; internal consistency was high (family α = 0.88, friends α = 0.90, and significant others α = 0.88).

***Stigma***

Attitudes toward people who die by suicide were measured using the Short Form of the Stigma of Suicide Scale (SOSS; Batterham et al., 2013b), which asks participants to rate how much they agree or disagree with words describing people who take their own lives. Sixteen items, e.g., “*irresponsible*,” “*lonely*,” “*noble*,” are rated on a 5-point scale from Strongly Disagree to Strongly Agree. Subscales of stigma, isolation/depression, and glorification/normalisation can be calculated by summing the relevant items for each subscale; internal consistency for the respective subscales was α = 0.83, α = 0.85, and α = 0.75.

Corrections have also been made to *Materials and Methods, Data Analytic Plan, Paragraph 1*. The corrected paragraph is shown below:

**Data Analytic Plan**

Missing data was dealt with using multiple imputation, as Little's MCAR test was non-significant, χ^2^(5) = 5.09, *p* = 0.405, and data was deemed to be most likely missing completely at random. A total of *m* = 68 imputations were generated based on 68% of cases having incomplete data (as suggested by White et al., 2010). Analyses were conducted using SPSS Version 27, which supports pooled analyses based on imputed datasets for several statistical tests; however, some analyses are not supported by this function. In such cases, parameter estimates were manually averaged across the 68 imputed datasets, an approach also taken by Jones et al. (2014) when dealing with imputed data in SPSS. Microsoft Excel 2013 was used to manually pool parameter estimates where necessary.

Additionally, corrections have been made to the *Results* section.

Corrections have been made to the *Results, Prevalence of Bereavement and Self-Harm at Baseline (T1)* and *Prevalence of Bereavement and Self-Harm at Follow-Up (T2)* sections. The corrected paragraphs are shown below:

**[RESULTS]**

**Prevalence of Bereavement and Self-Harm at Baseline (T1)**

A comparison of those who took part at baseline only and those who participated at both time points revealed no significant differences on most of the demographic or studied variables, apart from SES, family social support, and lifetime self-harm (those followed-up came from schools with a lower proportion of pupils receiving free school meals, reported lower family social support, and were more likely to report self-harm at baseline). Descriptive statistics of continuous study variables for all participants across all self-harm groups are shown in Table 1. At baseline (*n* = 185), 136 (73.51%) young people reported that someone among their immediate family and/or someone else close had died; 21 (11.35%) of which knew someone who had died by suicide (making up the suicide exposed group), while the remaining 115 (62.16%) people were exposed to a non-suicide death.

**Table 1:** Descriptive statistics for continuous scale variables for participants at both time points, within each self-harm group.

	**Total (*****M, SD*****)**		**Control (*****M, SD*****)**		**Ideation (*****M, SD*****)**		**Enactment (*****M, SD*****)**	
	**T1 (*n* = 185)**	**T2 (*n* = 115)**	**T1 (*n* = 103)**	**T2 (*n* = 56)**	**T1 (*n* = 44)**	**T2 (*n* = 26)**	**T1 (*n* = 38)**	**T2 (*n* = 33)**
Age	13.16 (1.49)	13.65 (1.52)	12.89 (1.42)	13.30 (1.40)	13.43 (1.49)	14.08 (1.67)	13.58 (1.57)	13.91 (1.49)
SES	14.29 (5.19)	13.29 (5.22)	14.96 (4.77)	13.50 (5.10)	12.24 (5.80)	12.41 (5.50)	14.83 (5.05)	13.60 (5.26)
Depression	7.72 (7.18)	8.57 (7.82)	4.16 (4.75)	3.03 (3.36)	9.71 (6.41)	10.00 (5.49)	15.07 (7.12)	16.85 (7.00)
Anxiety	6.78 (6.26)	8.08 (7.10)	3.91 (4.83)	3.41 (4.38)	9.41 (5.85)	10.73 (6.41)	11.48 (6.11)	13.91 (5.96)
Defeat	17.90 (15.07)	20.19 (16.72)	10.50 (8.87)	9.65 (8.85)	21.96 (14.30)	21.46 (10.01)	33.26 (16.11)	37.07 (17.11)
Entrapment	14.13 (14.68)	16.31 (16.67)	6.79 (8.57)	4.98 (6.59)	18.44 (13.55)	21.75 (13.64)	29.01 (16.01)	31.26 (17.03)
Adaptive coping	33.91 (8.79)	34.14 (9.13)	32.43 (8.56)	32.41 (10.21)	35.35 (9.52)	37.75 (6.95)	36.29 (7.89)	34.25 (7.99)
Maladaptive coping	21.96 (5.80)	22.08 (6.84)	19.13 (4.61)	17.97 (4.39)	24.31 (4.97)	24.79 (5.42)	26.93 (5.07)	26.93 (7.14)
Self-esteem	21.49 (6.10)	22.07 (6.50)	18.77 (4.65)	17.82 (4.36)	23.56 (6.11)	23.77 (3.54)	26.48 (5.55)	27.92 (6.24)
SS - family	5.67 (1.44)	5.51 (1.63)	6.16 (1.08)	6.31 (0.82)	5.37 (1.32)	5.53 (1.32)	4.71 (1.81)	4.14 (1.97)
SS - friends	5.24 (1.56)	5.31 (1.68)	5.45 (1.36)	5.40 (1.63)	5.04 (1.76)	5.56 (1.59)	4.92 (1.76)	4.96 (1.82)
SS - significant other	5.56 (1.50)	5.70 (1.48)	5.79 (1.30)	5.86 (1.3)	5.57 (1.48)	5.98 (1.41)	4.92 (1.86)	5.22 (1.72)
SOSS - stigma	2.10 (0.74)	1.92 (0.74)	2.15 (0.72)	2.03 (0.76)	2.13 (0.75)	2.19 (0.81)	1.91 (0.74)	1.52 (0.48)
SOSS - Iso/Dep	3.57 (1.04)	3.71 (0.98)	3.38 (1.06)	3.31 (1.03)	3.71 (0.91)	4.17 (0.85)	3.90 (1.01)	4.03 (0.70)
SOSS - Glo/Nor	2.52 (0.90)	2.61 (0.87)	2.48 (0.93)	2.58 (0.93)	2.73 (0.94)	2.41 (0.90)	2.40 (0.77)	4.03 (0.70)

*SES, socioeconomic status; SS, social support; SOSS, Stigma of Suicide Scale; Iso/Dep, isolation/depression subscale; Glo/Nor, glorification/normalisation subscale*.

38 (20.54%) pupils reported having ever engaged in self-harm behaviours during their lifetime (enactment group), while a further 44 (23.78%) reported past self-harm ideation with no history of behaviours (ideation group). Thus, the control group at baseline consisted of 103 (55.68%) individuals with no history of self-harm or suicidal thoughts or behaviours.

**Prevalence of Bereavement and Self-Harm at Follow-Up (T2)**

At follow-up, 81 participants of the T2 sample of *n* = 115 reported that someone among their immediate family and/or someone else close had died. Seventeen (14.78%) individuals overall reported knowing someone who had died by suicide (suicide exposed group), of which 10 (8.70%) were reported for the first time since T1. A further 66 (57.39%) individuals were exposed to a non-suicide death, with 13 (11.30%) reported for the first time since baseline. It is worth noting that two individuals responded ‘no' to the death of an immediate family member or anyone close, but ‘yes' to experiencing a suicide death of family or friends.

At follow-up, 33 (28.70%) adolescents reported ever engaging in self-harm, with 9 (7.83%) of these for the first time between Time 1 and Time 2. A further 26 (22.61%) individuals reported having experienced self-harm ideation (with no actions) at follow-up. The control group at T2 therefore comprised of 56 (58.70%) individuals who reported no history of self-harm ideation or behaviours at follow-up.

Corrections have also been made to *Results, Cross-Sectional Associations Between Motivational and Volitional Phase Variables and Self-Harm at Baseline (T1)*, sections *Motivational Phase Variables* and *Volitional Phase Variables*. The corrected sections are shown below:

**Cross-Sectional Associations Between Motivational and Volitional Phase Variables and Self-Harm at Baseline (T1)**

**Motivational Phase Variables**

A hierarchical multinomial logistic regression was conducted to examine whether motivational phase variables were associated with self-harm group status at baseline. In univariate analyses, those in the ideation group reported higher levels of entrapment (OR = 1.06, 95% CI = 1.01–1.11, *p* = 0.016), were more likely to employ maladaptive coping strategies (OR = 1.16, 95% CI = 1.04–1.30, *p* = 0.008), and report less available social support from family members (OR = 0.68, 95% CI = 0.50–0.93, *p* = 0.017) compared to controls, as expected (Table 2). Comparisons between the enactment group and controls showed similar patterns on the same variables as well as defeat (defeat: OR = 1.10, 95% CI = 1.04–1.18, *p* = 0.002; entrapment: OR = 1.09, 95% CI = 1.04–1.15, *p* = 0.001; maladaptive coping: OR = 1.22, 95% CI = 1.07–1.40, *p* = 0.003; family social support: OR = 0.57, 95% CI = 0.40–0.81, *p* = 0.002). As predicted, the ideation group did not differ from the enactment group on any motivational phase variable, apart from the enactment group being less likely to endorse glorifying/normalising beliefs about suicide (OR = 0.46, 95% CI = 0.25–0.84, *p* = 0.012).

**Table 2:** Univariate multinomial logistic regression of the association between motivational phase variables and self-harm group status at baseline (controlling for age, gender, depression, and anxiety).

**Motivational phase variable**		***B***	***SE***	**OR**	**95% CI for Odds Ratio**	***p***
**Defeat**						
Control	Ideation	0.06	0.03	1.06	1.00–1.12	0.049
Control	Enactment	0.10	0.03	1.10	1.04–1.18	**0.002**
Ideation	Enactment	0.04	0.03	1.04	0.99–1.10	0.117
**Entrapment**						
Control	Ideation	0.06	0.02	1.06	1.01–1.11	**0.016**
Control	Enactment	0.09	0.03	1.09	1.04–1.15	**0.001**
Ideation	Enactment	0.03	0.02	1.03	0.99–1.07	0.189
**Adaptive coping**						
Control	Ideation	0.02	0.02	1.02	0.97–1.07	0.411
Control	Enactment	0.04	0.03	1.04	0.98–1.11	0.157
Ideation	Enactment	0.02	0.03	1.02	0.97–1.08	0.426
**Maladaptive coping**						
Control	Ideation	0.15	0.06	1.16	1.04–1.30	**0.008**
Control	Enactment	0.20	0.07	1.22	1.07–1.40	**0.003**
Ideation	Enactment	0.05	0.06	1.05	0.94–1.18	0.396
**Self-esteem**						
Control	Ideation	0.11	0.05	1.12	1.01–1.24	0.035
Control	Enactment	0.12	0.06	1.12	0.99–1.27	0.066
Ideation	Enactment	0.00	0.06	1.00	0.90–1.12	0.970
**SS - family**						
Control	Ideation	−0.39	0.16	0.68	0.50–0.93	**0.017**
Control	Enactment	−0.57	0.18	0.57	0.40–0.81	**0.002**
Ideation	Enactment	−0.19	0.16	0.83	0.61–1.13	0.229
**SS - friends**						
Control	Ideation	−0.07	0.13	0.93	0.72–1.22	0.608
Control	Enactment	−0.03	0.16	0.97	0.71–1.33	0.850
Ideation	Enactment	0.04	0.15	1.04	0.78–1.39	0.793
**SS - significant other**						
Control	Ideation	−0.11	0.15	0.89	0.67–1.19	0.431
Control	Enactment	−0.28	0.17	0.75	0.54–1.04	0.089
Ideation	Enactment	−0.17	0.16	0.84	0.62–1.16	0.292
**SOSS - stigmatisation**						
Control	Ideation	−0.17	0.30	0.84	0.47–1.53	0.575
Control	Enactment	−0.65	0.38	0.52	0.25–1.09	0.084
Ideation	Enactment	−0.48	0.36	0.62	0.30–1.26	0.185
**SOSS–Iso/Dep**						
Control	Ideation	−0.06	0.23	0.95	0.60–1.50	0.813
Control	Enactment	−0.03	0.31	0.97	0.53–1.80	0.928
Ideation	Enactment	0.03	0.31	1.03	0.56–1.88	0.931
**SOSS - Glo/Nor**						
Control	Ideation	0.20	0.24	1.22	0.75–1.96	0.423
Control	Enactment	−0.59	0.32	0.55	0.30–1.03	0.060
Ideation	Enactment	−0.79	0.32	0.46	0.25–0.84	**0.012**

*Holm-Bonferroni corrections were applied; only the comparisons in bold remain significant at the adjusted significance level. SES, socioeconomic status. SS, social support; SOSS, Stigma of Suicide Scale; Iso/Dep, isolation/depression subscale; Glo/Nor, glorification/normalisation subscale*.

Significant univariate predictors associated with self-harm were entered into a multivariate analysis (Table 3), which found that three factors continued to be associated with self-harm group membership: ideation (OR = 1.15, 95% CI = 1.02–1.30, *p* = 0.022) and enactment (OR = 1.22, 95% CI = 1.04–1.42, *p* = 0.012) groups were more likely to report maladaptive coping compared to controls, as predicted; ideation (OR = 0.67, 95% CI = 0.47–0.94, *p* = 0.020) and enactment (OR = 0.60, 95% CI = 0.39–0.91, *p* = 0.016) groups were both more likely to report lower family social support compared to controls as predicted, and the enactment group were also less likely to hold glorifying/normalising beliefs about suicide than the ideation group (OR = 0.42, 95% CI = 0.22–0.80, *p* = 0.009).

**Table 3:** Multivariate multinomial logistic regression of the association between motivational phase variables and self-harm group status at baseline (controlling for age, gender, depression, and anxiety).

**Motivational phase variable**		***B***	***SE***	**OR**	**95% CI for Odds Ratio**	***p***
**Defeat**						
Control	Ideation	0.03	0.03	1.04	0.97–1.10	0.273
Control	Enactment	0.07	0.04	1.07	1.00–1.15	0.061
Ideation	Enactment	0.04	0.03	1.04	0.97–1.10	0.260
**Entrapment**						
Control	Ideation	0.03	0.03	1.03	0.97–1.08	0.336
Control	Enactment	0.04	0.03	1.04	0.97–1.10	0.281
Ideation	Enactment	0.01	0.03	1.01	0.96–1.07	0.764
**Maladaptive coping**						
Control	Ideation	0.14	0.06	1.15	1.02–1.30	**0.022**
Control	Enactment	0.20	0.08	1.22	1.04–1.42	**0.012**
Ideation	Enactment	0.05	0.07	1.05	0.92–1.21	0.450
**SS - family**						
Control	Ideation	−0.41	0.17	0.67	0.47 - 0.94	**0.020**
Control	Enactment	−0.51	0.21	0.60	0.39–0.91	**0.016**
Ideation	Enactment	−0.11	0.18	0.90	0.63–1.28	0.554
**SOSS - Glo/Nor**						
Control	Ideation	0.22	0.26	1.25	0.75–2.08	0.397
Control	Enactment	−0.66	0.35	0.52	0.26–1.04	0.062
Ideation	Enactment	−0.88	0.34	0.42	0.22–0.80	**0.009**

*Holm-Bonferroni corrections were applied; only the comparisons in bold remain significant at the adjusted significance level. SES, socioeconomic status; SS, social support; SOSS, Stigma of Suicide Scale; Glo/Nor, glorification/normalisation subscale*.

**Volitional Phase Variables**

A similar logistic regression analysis was conducted to examine volitional phase variables and their association with self-harm group status at baseline. Univariate analyses showed that ideation and enactment groups did not differ from controls on any variable. The ideation group differed from the enactment group only on family self-harm (OR = 0.17, 95% CI = 0.04–0.70, *p* = 0.014), where those who self-harmed were more likely to report this experience (Table 4). Neither experiencing a suicide nor a non-suicide death were associated with self-harm group membership. A multivariate analysis was not conducted as only one variable emerged as a significant predictor in this analysis.

**Table 4:** Univariate multinomial logistic regression of the association between volitional phase variables and self-harm group status at baseline (controlling for age, gender, depression, and anxiety).

**Volitional phase variable**		***B***	***SE***	**OR**	**95% CI for Odds Ratio**	***p***
**Suicide death**						
Control	Ideation	0.02	0.80	1.02	0.21–4.91	0.977
Control	Enactment	−1.18	0.74	0.31	0.07–1.31	0.111
Ideation	Enactment	−1.20	0.68	0.30	0.08–1.13	0.075
**Non-suicide death**						
Control	Ideation	0.14	0.44	1.15	0.49–2.71	0.744
Control	Enactment	0.32	0.51	1.37	0.50–3.74	0.538
Ideation	Enactment	0.17	0.50	1.19	0.45–3.16	0.729
**Family self-harm**						
Control	Ideation	1.16	0.79	3.20	0.69–14.93	0.139
Control	Enactment	−0.61	0.64	0.54	0.16–1.90	0.341
Ideation	Enactment	−1.77	0.72	0.17	0.04–0.70	**0.014**
**Friend self-harm**						
Control	Ideation	−0.67	0.45	0.51	0.21–1.24	0.137
Control	Enactment	−0.93	0.52	0.39	0.14–1.08	0.071
Ideation	Enactment	−0.27	0.50	0.77	0.29–2.03	0.594

*Holm-Bonferroni corrections were applied; only the comparisons in bold remain significant at the adjusted significance level*.

Corrections have also been made to *Results, Longitudinal Associations Between Motivational and Volitional Phase Variables and Self-Harm at Follow-Up (T2)*, sections *Motivational Phase Variables* and *Volitional Phase Variables*. The corrected sections are shown below:

**Longitudinal Associations Between Motivational and Volitional Phase Variables and Self-Harm at Follow-Up (T2)**

**Motivational Phase Variables**

A hierarchical multinomial logistic regression examined whether motivational phase variables were associated with life-time self-harm group 6-months later. In univariate analyses, participants in the ideation group were significantly more likely than controls to have reported self-harm ideation at baseline (Table 5a; OR = 0.08, 95% CI = 0.02–0.34, *p* = 0.001). Those in the enactment group differed from controls on family social support (OR = 0.53, 95% CI = 0.33–0.87, *p* = 0.011), stigmatising beliefs about suicide (OR = 0.27, 95% CI = 0.09–0.78, *p* = 0.016), glorifying/normalising beliefs about suicide (OR = 0.31, 95% CI = 0.12–0.76, *p* = 0.010), and self-harm ideation at baseline (OR = 0.05, 95% CI = 0.01–0.26, *p* = 0.001). The enactment group did not differ from the ideation group on any motivational phase variable.

**Table 5a:** Univariate multinomial logistic regression of the association between motivational phase variables and self-harm group status at follow-up (controlling for age, gender, depression, and anxiety).

**Motivational phase variable**		***B***	***SE***	**OR**	**95% CI for Odds Ratio**	***p***
**Defeat**						
Control	Ideation	0.03	0.04	1.03	0.96–1.10	0.475
Control	Enactment	0.02	0.04	1.02	0.94–1.10	0.696
Ideation	Enactment	−0.01	0.03	0.99	0.93–1.06	0.757
**Entrapment**						
Control	Ideation	0.07	0.04	1.07	1.00–1.14	0.053
Control	Enactment	0.07	0.04	1.07	1.00–1.16	0.055
Ideation	Enactment	0.01	0.03	1.01	0.95–1.06	0.873
**Adaptive coping**						
Control	Ideation	0.00	0.04	1.00	0.94–1.08	0.923
Control	Enactment	−0.07	0.04	0.93	0.86–1.01	0.091
Ideation	Enactment	−0.08	0.04	0.93	0.86–1.01	0.066
**Maladaptive coping**						
Control	Ideation	0.04	0.08	1.04	0.89–1.22	0.611
Control	Enactment	−0.01	0.09	1.00	0.84–1.18	0.952
Ideation	Enactment	−0.05	0.08	0.96	0.82–1.11	0.559
**Self-esteem**						
Control	Ideation	0.05	0.07	1.05	0.91–1.21	0.514
Control	Enactment	0.08	0.08	1.08	0.92–1.28	0.348
Ideation	Enactment	0.03	0.08	1.03	0.89–1.20	0.688
**SS - family**						
Control	Ideation	−0.17	0.23	0.85	0.54–1.32	0.466
Control	Enactment	−0.63	0.25	0.53	0.33–0.87	**0.011**
Ideation	Enactment	−0.47	0.22	0.63	0.41–0.96	0.034
**SS - friends**						
Control	Ideation	−0.01	0.19	0.99	0.69–1.44	0.972
Control	Enactment	0.09	0.23	1.10	0.70–1.71	0.687
Ideation	Enactment	0.10	0.20	1.10	0.74–1.64	0.628
**SS - significant other**						
Control	Ideation	−0.25	0.20	0.78	0.53–1.17	0.231
Control	Enactment	−0.52	0.22	0.60	0.39–0.93	0.022
Ideation	Enactment	−0.27	0.20	0.76	0.51–1.14	0.186
**SOSS - stigmatisation**						
Control	Ideation	−0.28	0.44	0.76	0.32–1.80	0.533
Control	Enactment	−1.32	0.55	0.27	0.09–0.78	**0.016**
Ideation	Enactment	−1.05	0.51	0.35	0.13–0.96	0.041
**SOSS - Iso/Dep**						
Control	Ideation	0.09	0.35	1.09	0.55–2.18	0.808
Control	Enactment	−0.14	0.41	0.87	0.39–1.95	0.741
Ideation	Enactment	−0.22	0.40	0.80	0.37–1.76	0.582
**SOSS - Glo/Nor**						
Control	Ideation	−0.55	0.38	0.58	0.27–1.21	0.145
Control	Enactment	−1.18	0.46	0.31	0.12–0.76	**0.010**
Ideation	Enactment	−0.63	0.40	0.53	0.24–1.17	0.118
**Self-harm ideation at T1**						
Control	Ideation	−2.51	0.73	0.08	0.02–0.34	**0.001**
Control	Enactment	−3.08	0.89	0.05	0.01–0.26	**0.001**
Ideation	Enactment	−0.56	0.83	0.57	0.11–2.90	0.498

*Holm-Bonferroni corrections were applied; only the comparisons in bold remain significant at the adjusted significance level. SES, socioeconomic status; SS, social support; SOSS, Stigma of Suicide Scale; Iso/Dep, isolation/depression subscale; Glo/Nor, glorification/normalisation subscale*.

Significant univariate predictors associated with self-harm were entered into a multivariate analysis (Table 5b), which found that three factors continued to be associated with self-harm group membership: the ideation group were less likely to report glorifying/normalising beliefs about suicide (OR = 0.19, 95% CI = 0.06–0.66, *p* = 0.009), and more likely to report self-harm ideation at baseline (OR = 0.03, 95% CI = 0.00–0.20, *p* < 0.001) compared to controls. In addition, the enactment group were less likely to hold stigmatising beliefs about suicide (OR = 0.18, 95% CI = 0.05–0.73, *p* = 0.016), less likely to hold glorifying/normalising beliefs about suicide (OR = 0.11, 95% CI = 0.03–0.46, *p* = 0.002), and more likely to have reported self-harm ideation at baseline (OR = 0.02, 95% CI = 0.00–0.22, *p* = 0.002), compared to controls.

**Table 5b:** Multivariate multinomial logistic regression of the association between motivational phase variables and self-harm group status at follow-up (controlling for age, gender, depression, and anxiety).

**Motivational phase variable**		***B***	***SE***	**OR**	**95% CI for Odds Ratio**	***p***
**SS - family**						
Control	Ideation	−0.09	0.28	0.91	0.53–1.59	0.747
Control	Enactment	−0.57	0.31	0.57	0.31–1.04	0.067
Ideation	Enactment	−0.47	0.23	0.62	0.39–0.99	0.043
**SOSS - stigmatisation**						
Control	Ideation	−0.78	0.57	0.46	0.15–1.41	0.173
Control	Enactment	−1.70	0.70	0.18	0.05–0.73	**0.016**
Ideation	Enactment	−0.92	0.55	0.40	0.14–1.17	0.094
**SOSS–Glo/Nor**						
Control	Ideation	−1.64	0.63	0.19	0.06–0.66	**0.009**
Control	Enactment	−2.20	0.72	0.11	0.03–0.46	**0.002**
Ideation	Enactment	−0.56	0.46	0.57	0.23–1.42	0.230
**Self-harm ideation at T1**						
Control	Ideation	−3.54	0.98	0.03	0.00–0.20	**<0.001**
Control	Enactment	−3.97	1.26	0.02	0.00–0.22	**0.002**
Ideation	Enactment	−0.43	0.99	0.65	0.09–4.56	0.668

*Holm-Bonferroni corrections were applied; only the comparisons in bold remain significant at the adjusted significance level. SES, socioeconomic status; SS, social support; SOSS, Stigma of Suicide Scale; Iso/Dep, isolation/depression subscale; Glo/Nor, glorification/normalisation subscale*.

**Volitional Phase Variables**

Another analysis was conducted to examine volitional phase variables and their association with self-harm group status prospectively. In univariate tests, none of the variables, including experiencing a suicide or a non-suicide death, emerged as significant predictors of self-harm group membership (Table 6). A multivariate analysis was therefore not required.

**Table 6:** Univariate multinomial logistic regression of the association between volitional phase variables and self-harm group status at follow-up (controlling for age, gender, depression, and anxiety).

**Volitional phase variable**		***B***	***SE***	**OR**	**95% CI for Odds Ratio**	***p***
**Suicide death**						
Control	Ideation	−2.02	1.41	0.13	0.01–2.12	0.153
Control	Enactment	−2.31	1.45	0.10	0.01–1.72	0.112
Ideation	Enactment	−0.29	0.82	0.75	0.15–3.74	0.725
**Non-suicide death**						
Control	Ideation	−0.30	0.64	0.74	0.21–2.57	0.635
Control	Enactment	−1.01	0.79	0.37	0.08–1.72	0.202
Ideation	Enactment	−0.71	0.74	0.49	0.12–2.08	0.336
**Family self-harm**						
Control	Ideation	−0.70	0.95	0.50	0.08–3.19	0.462
Control	Enactment	−1.44	0.96	0.24	0.04–1.55	0.134
Ideation	Enactment	−0.74	0.79	0.48	0.10–2.24	0.347
**Friend self-harm**						
Control	Ideation	0.18	0.65	1.20	0.34–4.25	0.776
Control	Enactment	−1.17	0.70	0.31	0.08–1.22	0.094
Ideation	Enactment	−1.35	0.67	0.26	0.07–0.97	0.044

*Holm-Bonferroni corrections were applied; only the comparisons in bold remain significant at the adjusted significance level*.

Overall, cross-sectional analyses showed that maladaptive coping, family social support and endorsing glorifying/normalising beliefs about suicide (motivational phase variables) and family self-harm (volitional phase variable) were significant predictors of self-harm group status. Longitudinally, endorsing stigmatising beliefs about suicide, endorsing glorifying/normalising beliefs about suicide, and self-harm ideation at baseline (motivational phase variables) predicted self-harm group at follow-up.

Lastly, corrections were also made to the *Discussion* section.

Corrections have been made to *Discussion, Paragraph 2*. The corrected paragraph is shown below:

**[DISCUSSION]**

Results partially supported the hypotheses both cross-sectionally and longitudinally. Although several variables (defeat, entrapment, maladaptive coping, family social support, and endorsing glorifying/normalising beliefs about suicide) predicted self-harm group membership in univariate analyses, only maladaptive coping, social support from family members and endorsing glorifying/normalising beliefs about suicide remained significant multivariate predictors within the motivational phase of the model. Family self-harm was the only predictor among the volitional phase variables to predict self-harm group cross-sectionally. Results of longitudinal analyses showed that endorsing stigmatising beliefs about suicide, endorsing glorifying/normalising beliefs about suicide, and self-harm ideation (motivational phase test) at baseline predicted self-harm group membership 6-months later, with none of the volitional phase variables emerging as significant predictors of prospective self-harm.

Corrections have also been made to *Discussion, IMV Model Psychological Variables as Predictors of Self-Harm, All Paragraphs*

**IMV Model Psychological Variables as Predictors of Self-Harm**

As predicted, maladaptive coping was found to be associated with self-harm group at baseline, and both ideation and enactment group participants differed from controls but not each other. This is in keeping with the large body of research that associates low levels of coping skills with suicidal thoughts and feelings (Gooding et al., 2015). Research particularly highlights that avoidant or emotion-focused (rather than problem-focused) coping styles are associated with self-harm in adolescents (Guerreiro et al., 2013), including the use of alcohol and drugs, behavioural disengagement and self-blame strategies, which were measured by the maladaptive coping subscale used in this study. The finding that only the maladaptive subscale was significant may reflect that self-harm might represent a coping style in itself; Laye-Gindhu and Schonert-Reichl (2005) suggest that self-harm is an emotion-focused strategy that serves to regulate affect. Indeed, research on motivations for self-harm among adolescents has found that getting relief from a terrible state of mind was the strongest predictor of self-harm (Rasmussen et al., 2016), which may account for the significant association found here.

Our cross-sectional findings reflect previous research (Kleiman and Liu, 2013; O'Connor and Nock, 2014) and theory (O'Connor, 2011; O'Connor and Kirtley, 2018) showing that levels of social support are significantly associated with suicide risk. In a recent large-scale study, Wan et al. (2019) found that lower social support was significantly associated with self-reported NSSI, suicidal ideation and suicide attempts among young people aged 10–20 years old. Our finding that only family social support was associated with self-harm group membership is consistent with Cheng and Chan (2007); using a translated version of the MSPSS, they found that the impact of family social support was stronger than that of friends in predicting suicidality among adolescents. Similarly, Tabaac et al. (2016) reported that social support from family and significant others was associated with suicidal ideation, but only family social support was associated with suicide attempts. They suggest that family members may represent a closer and more permanent source of support than other social groups, particularly for adolescents dealing with stressful life events.

As predicted, endorsing stigmatising beliefs about suicide significantly differentiated controls from enactment groups in longitudinal analyses, and glorifying/normalising beliefs differentiated controls from both ideation and enactment groups prospectively. It was found that higher levels of such beliefs were associated with being in the control group. The finding of a significant association at baseline in the motivational phase variable of glorifying/normalising beliefs about suicide was contrary to IMV model predictions, as ideation and enactment groups were not expected to differ. The ideation group were more likely to endorse glorifying or normalising beliefs about suicide than the enactment group at baseline. Previous research using the same SOSS measure (Batterham et al., 2013a) also showed that suicidal ideation was associated with greater glorification of suicide, as well as less stigma toward suicide, whereas suicide attempts were not associated with any attitude subscale (stigma, isolation/depression, or glorification/normalisation). One possible explanation for both cross-sectional and longitudinal findings is that individuals who self-harm are more likely to have been exposed to similar behaviours in others (Dhingra et al., 2015; Mars et al., 2019b), and increased exposure has been shown to reduce stigma (e.g., in relation to mental disorders; Jorm and Wright, 2008); in this study, experiencing self-harm of family members was indeed associated with self-harm group status cross-sectionally, which may account for the lack of an association with glorifying/normalising beliefs among the enactment group. Interestingly, self-harm group status was not associated with suicide bereavement. Given the small numbers of young people bereaved by suicide it was beyond the scope of this research to compare different bereavement groups. However, Bartik et al. (2015) found that those bereaved by suicide were more likely than the general population to perceive suicide as stigmatising and in glorifying or normalising terms, and less likely to attribute it to isolation and depression. Future research should therefore endeavour to assess how attitudes impact help-seeking among those who are suicide bereaved, to better understand the relationship between attitudes and self-harm.

The finding that baseline self-harm ideation predicted self-harm group at follow-up is consistent with past research (Ribeiro et al., 2016) and the theoretical assertion that ideation/intention is a proximal predictor of engagement in behaviours (O'Connor, 2011; O'Connor and Kirtley, 2018). We also found that self-harm among family members could predict self-harm group membership at baseline. In a UK population-based cohort study, Mars et al. (2019a) showed that exposure to family self-harm was a predictor of future suicide attempts among adolescents who reported suicidal thoughts (but not those who engaged in NSSI). O'Connor et al. (2009a) found that adolescents who engaged in repeat self-harm over a 6-month period were also significantly more likely to have family and friends who self-harmed than those who did not report self-harm; however, only family self-harm remained a significant predictor in multivariate analyses. These findings may be explained by familial transmission of suicidal behaviour (O'Connor et al., 2009a; Pitman et al., 2014), possibly through increased risk from shared environmental stressors or genetic factors, or transmission of psychopathology and impulsive aggression (Brent et al., 2002; Melhem et al., 2007). On the other hand, the finding that self-harm of friends did not predict self-harm group status here may also be attributed to a lack of statistical power, as numerous studies have suggested a role for social modelling of self-harm among non-family members. Self-harm among peers significantly predicted future suicidal behaviour in four large-scale studies across various countries (De Leo and Heller, 2008), where the sample sizes ranged from *n* = 731 to 11,572, depending on the study time point. This effect is observed in studies specifically with adolescents (Hawton et al., 2002; Doyle et al., 2015). Given the small sample in this study, further work is required to test this using a larger dataset.

Overall, some support for the IMV model was found. That several factors did not predict self-harm group membership cross-sectionally nor longitudinally may likely be the result of limited statistical power. The baseline self-harm groups consisted of 38 people in the enactment group, 44 in the ideation group, and 103 controls. At follow up, there were only 33 individuals in the self-harm enactment group, 26 in the ideation group, and 56 controls. While the sample sizes were deemed adequate for the analyses chosen, they may not have been sufficient to detect group differences, if these existed, where cell sizes were small (e.g., one third of cells had values of less than 5 in the suicide death and family self-harm variables at T2). Risk factors for self-harm can vary significantly over time and even within a day (Kashyap et al., 2015), so estimating future outcomes from measures taken 6 months earlier is particularly challenging, especially when using a small sample. It is also possible that the IMV model does not appropriately model the relationship between certain variables, or may not be applicable to young people in a Scottish context. Given the absence of an association between various established risk factors (including defeat and entrapment) and self-harm in multivariate analyses, additional research is needed to determine whether these findings hold with a larger sample, and ultimately whether the model requires further refinement. Future research should also examine the difference between internal and external entrapment; we refrained from exploring this due to the small sample size.

Corrections have also been made to *Discussion, Implications, Paragraph 1*. The corrected paragraph is shown below:

**Implications**

These findings offer an important contribution to the limited literature on adolescent bereavement experiences and their relation to self-harm. Results highlight that self-harm ideation and behaviours are prevalent among Scottish youth, and a large proportion of adolescents have also been bereaved or exposed to the death of someone close to them. Given the potential consequences of bereavement, and particularly suicide bereavement with its association to adverse outcomes, understanding the extent and nature of this experience among adolescents is essential. As a test of a theoretical model, support is promising for some aspects of the IMV model, in particular identifying stigmatising and glorifying/normalising beliefs about suicide, and self-harm ideation as predictors of future behaviours. At the same time, evidence which was not wholly consistent with regards to the role of other variables within the IMV model, such as the impact of experiences of suicide loss, requires further investigation with larger samples to assess their placement within the model. In addition to guiding future research and theory refinement, our findings have implications on targets for clinical interventions and postvention. Efforts aimed at enhancing healthy coping skills, increasing family cohesion and social support, and addressing beliefs and attitudes about suicide (such as viewing suicide as stigmatised or glorified), and targeting self-harm ideation before it becomes severe, may be especially effective.

Lastly, corrections have been made to Tables 1, 2, 3, 4, 5 and 6, and a new Table 5b has been added.

The authors apologize for these errors and state that they do not change the scientific conclusions of the article in any way. The original article has been updated.
